# The All-Data-Based Evolutionary Hypothesis of Ciliated Protists with a Revised Classification of the Phylum Ciliophora (Eukaryota, Alveolata)

**DOI:** 10.1038/srep24874

**Published:** 2016-04-29

**Authors:** Feng Gao, Alan Warren, Qianqian Zhang, Jun Gong, Miao Miao, Ping Sun, Dapeng Xu, Jie Huang, Zhenzhen Yi, Weibo Song

**Affiliations:** 1 Institute of Evolution & Marine Biodiversity, Ocean University of China, Qingdao 266003, China; 2 Department of Life Sciences, Natural History Museum, London SW7 5BD, UK; 3 Yantai Institute of Coastal Zone Research, Chinese Academy of Sciences, Yantai 264003, China; 4 College of Life Sciences, University of Chinese Academy of Sciences, Beijing 100049, China; 5 Key Laboratory of the Ministry of Education for Coastal and Wetland Ecosystem, Xiamen University, Xiamen 361102, China; 6 State Key Laboratory of Marine Environmental Science, Institute of Marine Microbes and Ecospheres, Xiamen University, Xiamen 361102, China; 7 Key Laboratory of Aquatic Biodiversity and Conservation, Institute of Hydrobiology, Chinese Academy of Sciences, Wuhan 430072, China; 8 Guangzhou Key Laboratory of Subtropical Biodiversity and Biomonitoring, School of Life Science, South China Normal University, Guangzhou 510631, China

## Abstract

The phylum Ciliophora plays important roles in a wide range of biological studies. However, the evolutionary relationships of many groups remain unclear due to a lack of sufficient molecular data. In this study, molecular dataset was expanded with representatives from 55 orders and all major lineages. The main findings are: (1) 14 classes were recovered including one new class, Protocruziea n. cl.; (2) in addition to the two main branches, Postciliodesmatophora and Intramacronucleata, a third branch, the Mesodiniea, is identified as being basal to the other two subphyla; (3) the newly defined order Discocephalida is revealed to be a sister clade to the euplotids, strongly suggesting the separation of discocephalids from the hypotrichs; (4) the separation of mobilids from the peritrichs is not supported; (5) Loxocephalida is basal to the main scuticociliate assemblage, whereas the thigmotrichs are placed within the order Pleuronematida; (6) the monophyly of classes Phyllopharyngea, Karyorelictea, Armophorea, Prostomatea, Plagiopylea, Colpodea and Heterotrichea are confirmed; (7) ambiguous genera *Askenasia*, *CyclotrichiumParaspathidium* and *Plagiocampa* show close affiliation to the well known plagiopyleans; (8) validity of the subclass Rhynchostomatia is supported, and (9) the systematic positions of Halteriida and Linconophoria remain unresolved and are thus regarded as *incertae sedis* within Spirotrichea.

The ciliated protists are a large and diverse group of microbial eukaryotes that are of central importance in the functioning of microbial food webs by mediating the transfer of organic matter and energy between different trophic levels^1^,^2^. Due to their short lifespan and unique biological characters (e.g., nuclear dimorphism, chromosomal fragmentation), they have attracted the attention of researchers in a wide range of disciplines including cytology, evolutionary biology and genetics^3^. Despite this attention, however, no broadly accepted hypothesis exists for the phylogenetic relationships within the Ciliophora. For example, two widely used classification systems, i.e. those of Corliss^4^ based mainly on morphological characters, and Lynn^5^ inferred mainly from ultrastructural characters and small subunit ribosomal DNA (SSU rDNA) sequences, are broadly consistent at class-level but differ widely at order- or family- levels. In recent years, investigations based on gene trees have been increasingly used to reconstruct ciliate phylogeny and have helped to resolve a number of phylogenetic problems^6^,^7^,^8^,^9^,^10^,^11^. Unfortunately, most of these investigations are based on sequence data from a single gene, namely SSU rDNA, although a few multi-gene phylogenetic studies have been performed on far more limited numbers of species^6^,^9^,^10^,^11^. To date, no molecular phylogenetic analysis has focused on a full-scale classification of the Ciliophora at the level of order/family.

Comprehensive taxon sampling coupled with gene-rich analyses are critical for resolving accurate phylogenies^12^. However, sampling and identifying targeted ciliate species/groups is very difficult and time-consuming due to their microscopic size. According to Lynn^5^, there are about 300 families and 57 orders of ciliates. In the present study, phylogenetic analyses were carried out based on sequence data from up to four gene markers in a total of 152 species representing 110 families and 55 orders. The main aim of this investigation was to resolve phylogenetic relationships among the principal ciliate groups including all available orders (excluding Cariacotrichida for which the SSU rDNA sequence is short and *in vivo* characters have yet to be reported).

## Results

**Figure 1:**
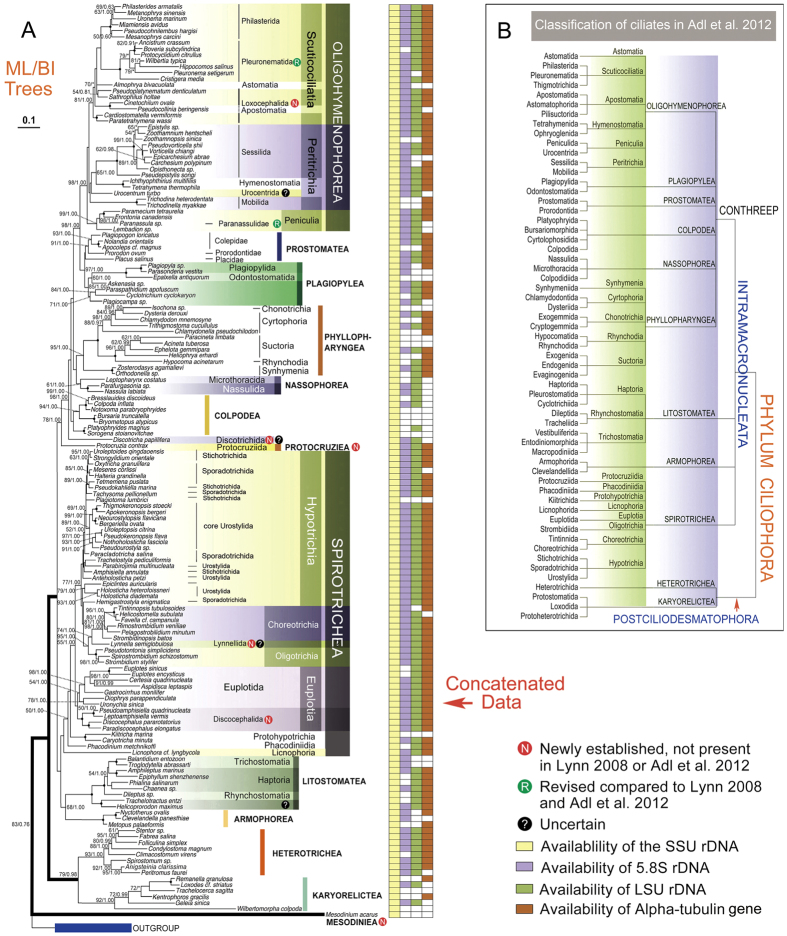
Phylogenetic tree (**A**) and classification (**B**) of the phylum Ciliophora. (**A**) Maximum likelihood (ML) tree reconstructed using 152 ciliates and concatenated genes (the SSU rDNA sequence is available for all the taxa whereas the 5.8S rDNA, LSU rDNA and alpha-tubulin gene sequences are available for only a subset of these taxa, Additional file 1: Table S2). Numbers at nodes represent the bootstrap values of maximum likelihood (ML) out of 1000 replicates and the posterior probability of Bayesian analysis (BI). Only bootstraps above 50% are shown. Fully supported (100%/1.00) branches are marked with solid circles. Asterisk (*) indicates disagreement between ML and BI analyses. The three main branches of ciliates are in bold. The scale bar corresponds to 10 substitutions per 100 nucleotide positions. (**B**) Classification scheme of phylum Ciliophora according to Lynn^5^ and Adl *et al.*^44^.

**Figure 2:**
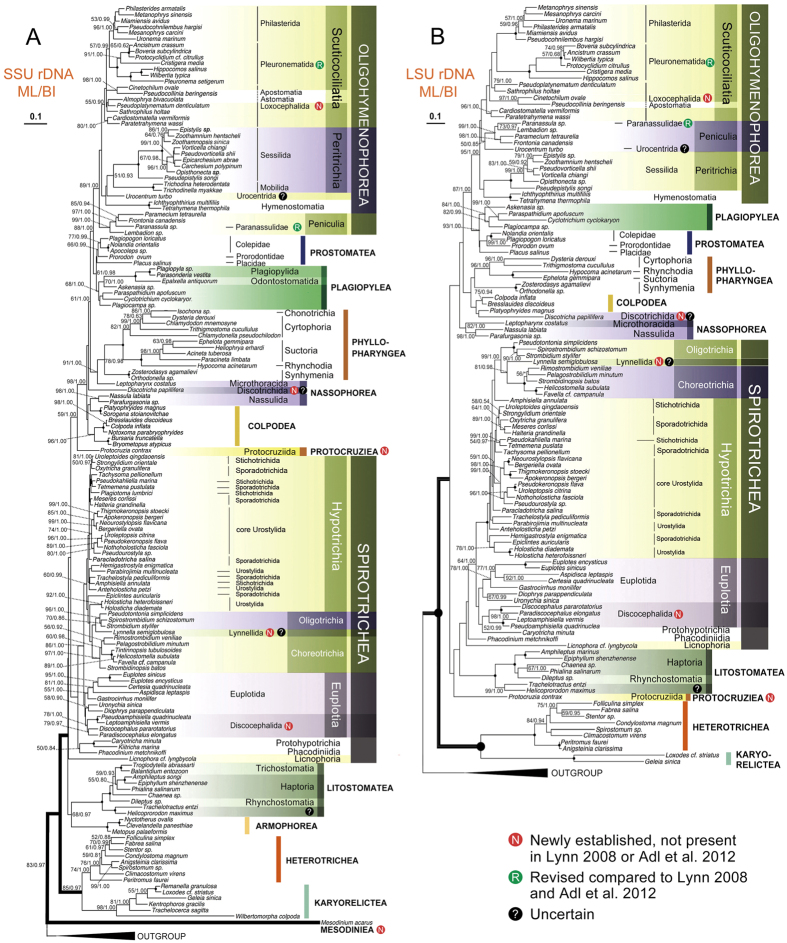
Maximum likelihood (ML) trees of the phylum Ciliophora based on the SSU rDNA ((**A**), 152 taxa) and LSU rDNA ((**B**), 118 taxa). Numbers at nodes represent the bootstrap values of maximum likelihood (ML) out of 1000 replicates and the posterior probability of Bayesian analysis (BI). Only bootstraps above 50% are shown. Fully supported (100%/1.00) branches are marked with solid circles. Asterisk (*) indicates disagreement between ML and BI analyses. The three main branches of ciliates are in bold. The scale bar corresponds to 10 substitutions per 100 nucleotide positions.

Concatenated, SSU rDNA, and LSU rDNA trees are topologically similar, and most of their nodes are well supported (Figs 1 and 2). By contrast, ITS1-5.8S-ITS2 rDNA and alpha-tubulin genes produce poorly supported topologies in both deep and crown nodes (Additional file 2: Figs S1–S6), so we do not describe these two trees in detail.

### Concatenated Tree

Both ML and BI analyses show a similar topology in the concatenated tree, that is, with three main groups are recognizable (Fig. 1A): (1) Mesodiniea, represented by the type genus *Mesodinium;* (2) subphylum Postciliodesmatophora, which comprises Karyorelictea and Heterotrichea, and; (3) subphylum Intramacronucleata, which comprises all other classes. Within the Postciliodesmatophora, the classes Heterotrichea and Karyorelictea is each recovered as a monophyletic assemblage. Within the Intramacronucleata, two major superclades are recovered, one comprising the classes Colpodea + Oligohymenophorea + Nassophorea + Plagiopylea + Prostomatea + Phyllopharyngea (CONthreeP), and the other comprising the classes: Litostomatea + Armophorea + Spirotrichea (SAL) (Fig. 1A).

Within the class Phyllopharyngea, the subgroup Subkinetalia (subclasses Cyrtophoria, Chonotrichia, Rhynchodia, and Suctoria), and the subclass Synhymenia, are both monophyletic and strongly supported (Fig. 1A).

In the class Nassophorea, the order Microthoracida clusters with the order Nassulida in the concatenated tree (61%ML, 1.00BI) (Fig. 1A). *Discotricha* is consistently placed as an early branching lineage within the ConThreeP superclade with high support (98% ML, 1.00 BI).

Protocruziidia, represented by the well-known genus *Protocruzia* and traditionally classified as a member of Spirotrichea^5^, groups with CONthreeP. With *Protocruzia* excluded, the class Spirotrichea is monophyletic (Fig. 1A), albeit with low support (<50% ML, <0.5 BI). Licnophorida occupies the basal position within the spirotrichean clade. Each of the other major subgroups, i.e. Euplotia, Protohypotrichia, Phacodiniida, Oligotrichia and Choreotrichia, forms a separate clade within the spirotrichean assemblage. By contrast, the subclass Hypotrichia is non-monophyletic with *Epiclintes*, *Holosticha* and *Hemigastrostyla* grouping with the choreotrichs and oligotrichs. Furthermore, groupings within the three hypotrich orders (Sporadotrichida, Stichotrichida, and Urostylida) are ambiguous. For example, six species of the order Stichotrichida occupy five separated clades; seven species of the order Sporadotrichida occupy six different clades; and although eight out of 13 species of Urostylida form a moderately supported clade (89% ML, 1.00 BI), the other five species occupy three separate clades. Within the Euplotia, the order Euplotida is monophyletic whereas the order Discocephalida clusters with *Pseudoamphisiella*.

As expected, the subclasses Oligotrichia and Choreotrichia, are both monophyletic with high support (Fig. 1A). The newly proposed order Lynnellida clusters with Choreotrichia, forming a group that is sister to the subclass Oligotrichia. The subclass Choreotrichia is comprised of two groups, the tintinnids and the aloricate choreotrichians.

The class Litostomatea consists of three subclasses, Haptoria, Trichostomatia and Rhynchostomatia. Haptoria is paraphyletic in all the gene trees (Figs 1 and 2), with the Trichostomatia (represented by *Balantidium* and *Troglodytella*) nesting within it and *Helicoprorodon* clustering with *Trachelotractus*, forming an early-branching lineage of litostomateans.

The Oligohymenophorea forms a maximally supported clade and comprises six subclasses (Fig. 1A). Both the subclasses Apostomatia and Astomatia, each of which is represented by a single sequence, nest within the scuticociliate assemblage rendering the Scuticociliatia polyphyletic. The scuticociliate order Philasterida is monophyletic. By contrast, the traditional scuticociliate order Pleuronematida sensu Lynn 2008, represented by five genera and four families, is not monophyletic with the thigmotrichids consistently nesting within it (100% ML, 1.00 BI). The order Loxocephalida, represented by five genera and two families, is paraphyletic with *Pseudoplatynematum* and *Sathrophilus* clustering with the Philasterida/Pleuronematida + Astomatia assemblage, *Cardiostomatella* and *Paratetrahymena* forming a separate clade that is basal to rest of the scuticociliates (100% ML, 1.00 BI), and *Cinetochilum* clustering with the subclass Apostomatia (100% ML, 1.00 BI).

The subclass Peritrichia comprises two orders, Sessilida and Mobilida. Although each order is monophyletic, the Sessilida groups with the subclass Hymenostomatia and the family Urocentridae (subclass Peniculia), rather than with the Mobilida, rendering the Peritrichia non-monophyletic.

The subclass Peniculia is represented by five families and five genera. Four of these genera (*Paramecium*, *Frontonia*, *Lambadion*, and *Paranassula*) form a well-supported clade that is basal to the rest of the Oligohymenophorea (Fig. 1A). The fifth genus, *Urocentrum* (family Urocentridae), occupies a position between the hymenostomes and the peritrich order Mobilida.

A close relationship between the classes Prostomatea and Plagiopylea is well supported (97% ML, 1.00 BI). The Prostomatea-Plagiopylea group (hereafter referred to as the PP clade) is sister to the class Oligohymenophorea with high support (98% ML, 1.00 BI), forming a three-class metaclade (Fig. 1A).

The class Prostomatea is represented by one order, namely Prorodontida, which is paraphyletic. The monophyletic family Colepidae (represented by *Apocoleps, Nolandia* and *Plagiopogon*) is closely related to Prorodontidae (represented by *Prorodon*) with variable support (91%ML, 1.00BI). The family Placidae (represented by *Placus*) is basal to the Colepidae-Prorodontidae. The prostome family Plagiocampidae (represented by *Plagiocampa*), however, clusters with two genera of uncertain taxonomic affiliation, *Cyclotrichium* and *Paraspathidium* (see below).

The class Plagiopylea is formed by the well-known plagiopyleans and four ambiguous genera, *Plagiocampa*, *Paraspathidium*, *Cyclotrichium* and *Askenasia*. The former three form a moderately supported sister group (71%ML, 1.00BI) to *Askenasia* and the well-known plagiopyleans (85% ML, 1.00 BI).

### Topological Difference between SSU rDNA and Concatenated Trees

Within the class Spirotrichea, the genus *Hemigastrostyla* is not sister to oligotrichs in the SSU rDNA tree (Fig. 2A), whereas this is the case in the concatenated tree (Fig. 1A), and the euplotid family Uronychiidae clusters with Discocephalida rather than Euplotida (Fig. 2A).

Within the class Oligohymenophorea, *Cristigera* groups with other cyclidiids and thigmotrichids with moderate to high support in the SSU rDNA tree (91% ML, 1.00 BI), rather than branching before all the other pleuronematids and thigmotrichids. Furthermore, in contrast to the concatenated tree, the monophyly of the subclass Peritrichia is supported in the SSU rDNA tree, with the sessilids and mobilids clustering together.

The subclass Licnophoria, represented by *Licnophora*, branches relatively early in the SSU rDNA tree and is basal to all groups except the assemblages Litostomatea-Armophorea and the Heterotrichea-Karyorelictea (Fig. 2A) vs. sister to Spirotrichea in the concatenated tree (Fig. 1A).

The two orders, Microthoracida and Nassulida, which are believed to be the members of the class Nassophorea, are placed in separate clades: the former branches in a position that is sister to the class Phyllopharyngea whereas the latter clusters with the colpodean genera *Platyophrides* and *Sorogena* (Fig. 2A) vs. form one clade in the concatenated tree (Fig. 1A).

### Topological Difference between LSU rDNA and Concatenated Trees

Various taxa are located in different positions in the LSU rDNA tree compared to the concatenated tree (Figs 1A and 2B). These include: *Protocruzia*, which branches within the SAL clade (vs. with the CONthreeP clade); the genera *Amphisiella*, *Trachelostyla*, *Parabirojimia* and *Anteholosticha*, all of which are located outside (vs. within) the main hypotrich clade; *Caryotricha*, which is nested within the Discocephalida (vs. basal to Spirotrichea); the order Lynnellida, which is more closely related to the subclass Oligotrichia than to the subclass Choreotrichia; *Wilbertia*, which clusters with the thigmotrichids (vs. with the pleuronematid *Hippocomos*); and *Urocentrum*, which clusters with the peniculids (vs. with the peritrichs and hymenostomes). Furthermore, in the SSU rDNA tree the basal clade within the class Oligohymenophorea is that comprising Peritrichia and Hymenostomatia whereas in the concatenated tree the Peniculia is basal.

## Discussion

### Relationships and Arrangement within the Phylum Ciliophora

**Figure 3:**
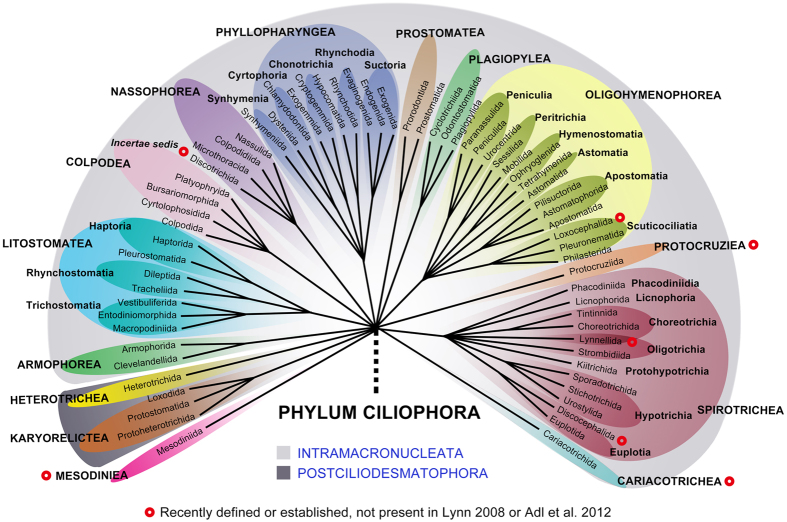
Systematic scheme for the phylum Ciliophora suggested by the present and previous works. The order Discocephalida was established by Wicklow^47^, and revised to contain two suborders Discocephalina and Pseudoamphisiellina by Miao *et al.*^45^. The order Discotrichida was established in Fan *et al.*^103^. The order Loxocephalida was originally proposed by Jankowski^18^ and was confirmed by Li *et al.*^66^, Gao *et al.*^60^, Zhang *et al.*^62^, etc. The order Lynnellida was established by Liu *et al.*^55^. The class Cariacotrichea was established by Orsi *et al.*^122^. The order Mesodiniida was resurrected and the class Mesodiniea was established by Chen *et al.*^17^. The classes Protocruziea and Licnophoriea are defined in the present study.

**Figure 4:**
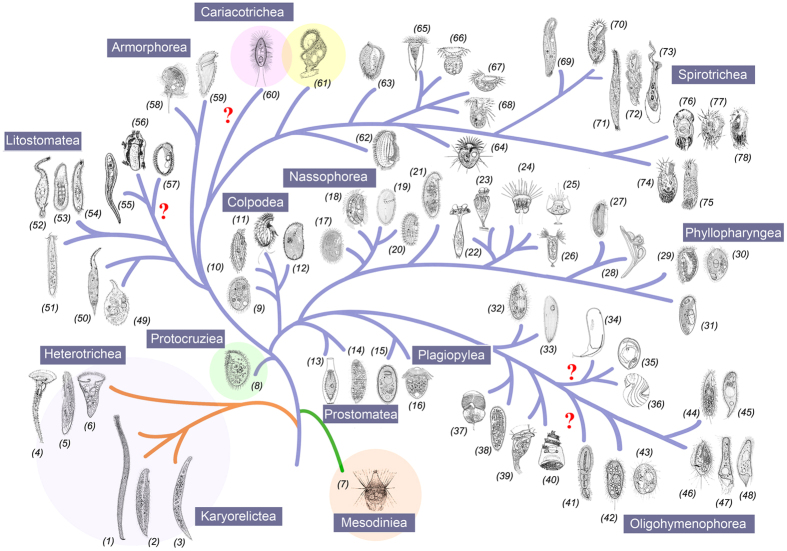
Hypothetical evolution of ciliated protozoa based on both morphological and molecular data to show the relationship and the positions of the taxa at order level. (1–8), (14–17), (21), (24–26), (29–32), (36), (38), (39), (42–54), (58), (61), and (63–78) are from the present authors. (9) is from Bardele *et al.*^123^. (10–13), (19), (22–23), (27–28), (33–35), (37), (40–41), (55–56), (59), and (62) are from Corliss^4^. (18) is from Foissner *et al.*^124^, (20) is from Foissner *et al.*^125^. (57) is from Dehority^126^. (60) is from Orsi *et al.*^122^.

It is widely accepted that the phylum Ciliophora comprises two main groups, the subphyla Intramacronucleata and Postciliodesmatophora (Figs 3 and 4), although relationships among the constituent classes are generally not well resolved due to low support values in gene trees^11^,^13^,^14^,^15^. In the concatenated and LSU rDNA trees (Figs 1A and 2B), two main groups could be recognized within the subphylum Intramacronucleata: one group has six classes, namely Colpodea, Oligohymenophorea, Nassophorea, Phyllopharyngea, Plagiopylea and Prostomatea (CONthreeP); the other main group comprises three morphologically diverse classes, Spirotrichea, Armophorea and Litostomatea (SAL). It is noteworthy, however, that whereas both Intramacronucleata and Postciliodesmatophora could be defined by morphological differences^5^, there are no strong morphological synapomorphies for either CONthreeP or SAL. Nevertheless, the clade comprising Armophorea and Litostomatea is supported by some morphological and morphogenetic synapomorphies, which unite them into a new infraphylum, Lamellicorticata^16^.

The subphylum Postciliodesmatophora comprises two classes, the Heterotrichea and Karyorelictea, both of which were recovered as monophyletic groups in our analyses. This is consistent both with previous phylogenetic analyses^15^,^17^ and with morphological data^5^.

The systematics of the family Mesodiniidae Jankowski in Small & Lynn, 1985, represented in the present study by the genus *Mesodinium*, has long been problematic. Traditionally it has been assigned to one of two orders within the subclass Haptoria, i.e. Haptorida^4^,^5^,^18^,^19^,^20^ or Cyclotrichida^5^. Puytorac^19^ established the order Mesodiniida for the family Mesodiniidae, however this taxon has not been widely accepted. Nevertheless, the systematic placement of the mesodiniids is ambiguous given that they invariably branch very deep in a range of gene trees and, based on their unique morphological features, they are usually completely isolated from other groups^17^,^21^,^22^. It has recently been suggested that the order Mesodiniida should be resurrected and that the new class Mesodiniea, be established for this taxon^17^. In the present study, Mesodiniea is consistently basal to all other ciliate lineages thus supporting its validity as a separate class and suggesting that it may even be separated at subphylum level (Figs 1A and 2A). However, the class Mesodiniea was not monophyletic in phylogenomic analyses based on 127 genes^9^. More data are therefore needed in order to validate this class.

### Relationships within Heterotrichs and Traditionally Related Groups

Ciliates with a non-specialized somatic ciliature and an adoral zone of membranelles have long been classified as heterotrichs^18^,^20^. In recent decades, some “traditional” heterotrichs such as armorphoreans, *Licnophora*, *Phacodinium*, *Protocruzia*, and *Plagiotoma* have been excluded based on ultrastructural and/or molecular data^23^,^24^,^25^. As a result of these findings, Lynn^5^ assigned the “true” heterotrichs to the class Heterotrichea, a decision that is firmly supported by the current analyses.

According to Lynn^5^, Phacodiniidia Small and Lynn, 1985 and Protocruziidia Puytorac *et al.*^25^ are subclasses of the class Spirotrichea. Each contains only a single genus. Hitherto, the systematic positions of these two subclasses have never been satisfactorily resolved. Both are usually placed within the class Spirotrichea despite the fact that neither has a replication band during macronuclear DNA replication, an important apomorphy for the Spirotrichea^5^,^20^,^26^,^27^. In the case of Protocruziidia, other evidence casting doubt on its affiliation to the Spirotrichea includes its infraciliature (non-specialized somatic ciliature, non-differentiation of the ventral-dorsal sides, etc)^27^ and its process of ontogenesis which is a mixture of parakinetal and scuticokinetal modes^28^. Although some early molecular studies suggested that *Protocruzia* could be related to Spirotrichea^24^,^29^,^30^, this finding has been repeatedly rejected by recent studies which invariably conclude that it is not a member of the class Spirotrichea^11^,^31^. Li *et al.*^31^ suggested that *Protocruzia* represents a separate class, although they failed to define this taxon. Thus, the new class Protocruziea n. cl. is formally established here, based on the subclass Protocruziidia which contains a single order, Protocruziida Jankowski in Small & Lynn, 1985, a single family, Protocruziidae Jankowski, 1980, and a single genus, *Protocruzia* de Faria, da Cunha and Pinto, 1922, with the characters diagnosed for its subclass and the order^5^. The current analyses reveal that Protocruziea n. cl. belongs to neither of the main sub-groups of Intramacronucleata (CONthreeP or SAL) but instead occupies a position between the two as sister to the CONthreeP/Discotrichida assemblage (Figs 1A and 2A).

#### Protocruziea de Puytorac *et al.* 1987 n. cl

##### Diagnosis

Body small-sized, bilaterally flattened; somatic ciliature typically with dikinetids on both left and right sides; extrusomes present; adoral zone with several membranelles on left of dominant oral region; paroral membrane composed of dikinetids; stomatogenesis in mixokinetal mode; nuclear apparatus as a cluster of similar-sized nuclei with paradiploid macronuclei surrounding one or more micronuclei; free-swimming in marine and brackish water habitats; one order.

##### Type order

Protocruziida Jankowski, 1980 The genus *Phacodinium* is another highly questionable taxon regarding its phylogenetic position having long been regarded as a heterotrich, then as having affiliations to the hypotrichs and finally as being an intermediate form between the heterotrichs and hypotrichs^32^. In Lynn & Small’s system^33^, it was treated as a *sedis mutabilis* and assigned to the order Phacodiniida. The first molecular investigation based on SSU rDNA sequence data concluded that Phacodiniida is basal within Spirotrichea and suggested the establishment of a new subclass Phacodiniidia^24^. The present study supports this finding with Phacodiniidia occupying a basal position within the Spirotrichea.

*Licnophora*, for which the subclass Licnophoria Corliss, 1957 was established, is characterized by its highly specialized ciliature, unique morphology (e.g. presence of the adhesive disc with concentric kinetal rings) and the unusual pattern in ontogenesis with the cell undergoing a highly modified form of homothetogenic fission, i.e., a type of parallel division producing two daughter cells that develop alongside each other, similar to that in peritrichs^4^,^28^. Lynn^5^ assigned Licnophoria to the class Spirotrichea, although this placement has been repeatedly contradicted by molecular studies which indicate that it does not consistently cluster with spirotricheans^15^,^17^,^34^. Regarding its unique morphology that is unlike that of any spirotrichean, Licnophoria may represent a separate class that is sister to Spirotrichea. However, as molecular data are available for only a few species and complete information on morphogenesis remain unclear, we treat Licnophoria as *incertae sedis* within the SAL group.

### Phylogeny of Hypotrichia sensu str

The subclass Hypotrichia *s.l.* (formerly Stichotrichia) is one of the most morphologically diverse group of ciliates and has been subject to numerous phylogenetic analyses^6^,^35^,^36^,^37^,^38^. This has resulted in the development of at least ten systematic systems^4^,^5^,^19^,^20^,^39^. In the Lynn’s system^5^, three orders of hypotrichs were recognized, namely Stichotrichida, Urostylida and Sporadotrichida, based mainly on the ventral ciliary pattern and partly on morphogenetic features. Until now, however, classification within this subclass remained unclear^6^,^35^,^36^,^37^,^38^.

In common with previous investigations, the multi-gene analyses reported here failed to separate the Stichotrichida and Sporadotrichida, the clustering patterns of these two orders being ambiguous due to low support values in all trees. This indicates that the pattern of the ventral ciliature, i.e. in specific, localized frontal and ventral groups in Sporadotrichida vs. in one or more linear longitudinal files in Stichotrichida, which is used as a main apomorphy at order level^5^, might be a result of convergent evolution. As in previous phylogenetic analyses^6^,^35^,^36^, the order Urostylida is non-monophyletic, consisting of monophyletic “core urostylids” and others (Figs 1 and 2). It is also noteworthy that classifications of hypotrichous orders based mainly on morphological characters are distinct from each other and none is completely consistent with trees based on molecular data^4^,^5^,^39^,^40^. Thus, it seems likely that some morphological characters regarded as apomorphies at order level might be plesiomorphies.

### Systematic Position of the Subclass Protohypotrichia

The subclass Protohypotrichia, which contains a single order Kiitrichida, was established in 2009 based on both ontogenetic and molecular information^41^. Historically, the members of this group were believed to be a primordial assemblage within the Hypotrichia or ancestral forms of euplotids^4^,^19^,^20^,^33^. This hypothesis has received increasing support, both by the addition of more gene sequence data^34^,^41^ and by the recognition of various unique morphological and morphogenetic characters^42^,^43^. In the present study, the addition of newly sequenced LSU rDNA, 5.8S rDNA, and alpha-tubulin genes invariably resulted in the protohypotricheans clustering in a well-supported clade that is basal to the hypotrichs and euplotids (Figs 1A and 2). Thus all available evidence, molecular, morphological and ontogenetic, indicates that the Protohypotrichia should be recognized as a distinct group at subclass rank that is ancestral both to the hypotrichs and to the euplotids.

### Systematics of the Subclass Euplotia and Related Groups

Based on previous studies and the results of the present work, we accept the system proposed by Adl *et al.*^44^ that the assemblage comprising the euplotids and discocephalids represents a distinct taxon at subclass level, namely Euplotia. Previously these two subgroups were treated as two orders (Euplotida and Discocephalida) in different subclasses^33^, or as suborders within the order Euplotida^5^. In the present study, the monophyly of Euplotida is recovered in trees inferred both from LSU rDNA gene sequence data alone (Fig. 2B) and from concatenated data of four genes (Fig. 1A), although the support values are not high. In the SSU rDNA tree the discocephalids nest within the Euplotida assemblage. The discocephalines are found only in marine biotopes and are characterized by their cephalized body shape. The group most closely related to Discocephalina is generally thought to be Pseudoamphisiellina, this conclusion being based both on morphological characters (e.g., cephalized body shape, highly developed fiber system connecting the cirri, generally two clearly separated ventral rows, and highly developed transverse cirri) and ontogenetic characters (e.g., the unique formation of the ventral rows during morphogenesis)^45^,^46^,^47^,^48^. This finding is supported by the present study, the sister relationship between these two groups being consistently recovered in all trees (Figs 1 and 2). We also propose to resurrect the order Discocephalida, originally established by Wicklow^47^, to contain two subgroups, namely Discocephalina and Pseudoamphisiellina^45^. Discocephalids have been assigned to a range of different groups^4^,^19^,^25^,^33^,^47^. Previous studies based both on ontogenetic^46^,^47^ and molecular data^45^,^49^ indicate that the discocephalids are clearly separated from the hypotrichs s. str., and probably represent an independent lineage at order level that is intermediate between the euplotids and other groups within the Spirotrichea. This hypothesis is firmly supported by the present study.

### New Understanding of the Oligotrichs s.l

Traditionally, the loricate (tintinnids) and non-loricate (oligotrichids) oligotrichs were considered to be sister groups^50^. Only in last three decades have they been assigned to separate subclasses, the Choreotrichia and Oligotrichia, respectively. Choreotrichians are characterized by having a closed AZM and the group includes both tintinnids and some non-loricate forms, e.g. the strobilidids and related taxa. By contrast, oligotrichians have an open AZM^5^,^33^,^44^,^51^,^52^. The separation of these two subclasses is supported by the present study.

The family Lynnellidae was established by Liu *et al.*^53^ for the genus *Lynnella*, which shares some morphological features with both oligotrichians and choreotrichians. In a previous study based on analyses of all available data, i.e. morphological and molecular characters, Li *et al.*^54^ concluded that members of the Lynnellidae are intermediate forms between the Choreotrichia and Oligotrichia. Recently, Liu *et al.*^55^ established the order, Lynnellida for the Lynnellidae. The present study broadly supports these findings with Lynellida either sister to the oligotrichians (Fig. 2A) or sister to choreotrichians (Figs 1A and 2B).

The order Halteriida, represented by the genus *Halteria*, is a unique group that has long puzzled taxonomists^4^,^5^,^19^,^33^,^44^. Based on their morphology and pattern of morphogenesis, halteriids share similarities with the oligotrichs *sensu lato* but differ from the hypotrichs^56^,^57^. However, the SSU rDNA gene sequence data suggest that Halteriida might be a member of the hypotrichs/stichotrichs, possibly belonging to the oxytrichids, a highly specialized group of hypotrichs with very conservative modes of stomatogenesis^24^,^58^. Given this conflicting evidence, we believe that the Halteriida should be regarded as *incertae sedis* within Spirotrichea.

### Phylogeny of Scuticociliates and Closely Related Taxa

Due to their small size and similar morphologies and ciliary patterns, scuticociliates are one of the most ambiguous groups of ciliates^4^,^5^,^59^,^60^,^61^,^62^. According to Lynn^5^, the subclass Scuticociliatia contains three orders: Philasterida, Pleuronematida, and Thigmotrichida. Although Philasterida is a well-outlined lineage, analyses of gene sequences data have challenged the monophyly of both Pleuronematida and Thigmotrichida^63^,^64^. Indeed as more data have accumulated, the thigmotrichids are often nested within the Pleuronematida, close to the cyclidiids^60^,^65^. This is broadly consistent with Puytorac^19^ who regarded the thigmotrichids as a suborder within the order Pleuronematida. A fourth order of scuticociliates, Loxocephalida, was originally proposed by Jankowski^18^ to contain certain *Cinetochilum*-like taxa which were previously assigned in the order Philasterida. Loxocephalida has been repeatedly recovered as a basal group to the core scuticociliates^66^. Present analyses indicate that Loxocephalida is a polyphyletic assemblage that is most closely related to Astomatia and Apostomatia, and clearly support its separation from Philasterida (Figs 1 and 2). Nevertheless, further studies are needed in order to clarify the systematics of the loxocephalids.

The Astomatia and Apostomatia are two specialized subclasses within the class Oligohymenophorea^5^,^67^,^68^. Astomes are endosymbionts typically found in the digestive tract of annelids, especially oligochaetes, and entirely lack an oral apparatus^19^. By contrast, apostomes are usually found as epibionts of marine and brackish water crustaceans, and have highly modified oral structures and polymorphic life cycles^69^. Analyses based on molecular data consistently reveal both groups to be closely related to the scuticociliates^60^,^62^. Present analyses show that either astomes or apostomes are more closely related to the loxocephalids than to the core scuticociliates, which is consistent with previous studies^60^,^62^,^70^. A reasonable hypothesis could be that they may be derived from loxocephalid-like ancestral lineages, their highly specialized morphologies being a result of adaptation to their symbiotic life styles^60^,^62^.

### Phylogeny of Peniculia and Hymenostomatia

The peniculians are characterized by their three oral polykinetids aligned longitudinally in the oral cavit 5,19,33,71,72,73. The present phylogenetic analyses are consistent with previous studies in recovering the peniculians as a group that occupies a basal position within the olighymenophorean assemblage and is most closely related to the hymenostomatians and peritrichs^74^,^75^.

The hymenostomatians are characterized by having a well-defined buccal cavity with a paroral membrane, which may be unciliated and reduced, and typically three oral polykinetids^5^. In keeping with previous arrangements^19^,^33^, Lynn^5^ divided the subclass Hymenostomatia into two orders, Tetrahymenida and Ophryoglenida, represented by the well-known model organisms *Tetrahymena* and *Ichthyophthirius*, respectively. The findings of the present study are consistent with the above assignments and with previous studies^76^, supporting the monophyly of Hymenostomatia and its two orders.

Urocentrids have traditionally been regarded as a family within the subclass Peniculia^4^, although Puytorac *et al.*^25^ elevated them to the rank of order, which was accepted in later classification schemes^5^,^19^. However, both morphological (e.g. distinctive girdle of somatic cilia, somatic kinetids only as monokinetids with broad, tangential transverse ribbon; somatic extrusomes as mucocysts, etc.) and molecular evidence reveal that the urocentrids are divergent from all other typical peniculines and the position of the urocentrid assemblage is unstable in gene trees^4^,^74^,^77^. Based on the present findings and morphological data, classifying the urocentrids as an order is acceptable, however the phylogenetic position of the order Urocentrida is uncertain.

### The Monophyly of the Traditional Peritrichs

Historically, peritrich ciliates were considered to be a well-defined group comprising two orders: Sessilida and Mobilida^4^,^33^. Recent molecular analyses, however, have challenged this arrangement suggesting that the molecular and morphological information are not always congruent^78^,^79^,^80^,^81^,^82^,^83^,^84^,^85^. Based on SSU rDNA sequence data, the monophyly of the Peritrichia s. l. was considered sufficiently doubtful for the recognition of the mobilids as a separate subclass from an entirely sessilid subclass Peritrichia s. str., a decision subsequently supported by alpha-tubulin gene trees^86^. It has recently been suggested that support for and against monophyly of the peritrichs s.l. depends on methods of alignment, and methods of masking ambiguously aligned nucleotide positions^87^. In the present study, with the inclusion of additional sequence data, the concatenated tree and single-gene trees result in conflicting findings concerning the relationship between the sessilids and mobilids. As shown in Figs 1 and 2, species of the two lineages were recovered as a monophyletic group in the SSU rDNA tree whereas they are separated in the concatenated tree, albeit with very low support values. However, sessilids and mobilids show a close relationship based on the morphological and the morphogenetic evidence which exclude them from all other non-peritrich groups^5^,^28^,^86^,^88^,^89^,^90^,^91^. Therefore, we do not believe there is sufficient evidence for the non-monophyly of the peritrichs s. l. or for the separation of the sessilids and mobilids at subclass level. Consequently we support the continued recognition of the subclass Peritrichia sensu Lynn 2008 and its two orders, Sessilida and Mobilida.

### Phylogenetic Relationship within Litostomatea

The class Litostomatea has been traditionally rather poorly defined as having an apically positioned cytostome, uniform somatic ciliation and a non-distinct oral apparatus^4^,^5^,^33^,^92^. Lynn^5^ recognized two subclasses, Haptoria and Trichostomatia. A third subclass, Rhynchostomatia, was recently established by Vd’ačný *et al.*^93^. Recent molecular phylogenetic analyses, however, do not provide unambiguous support for the monophyly of Haptoria, with several of its members grouping with Trichostomatia, and the haptorid genera *Helicoprorodon* and/or *Trachelotractus* occupying a basal position within the class Litostomatea^94^. Our analyses with additional sequences also failed to recover the subclass Haptoria as a monophyletic group. In addition, for the first time, we reveal the close phylogenetic relationship between *Helicoprorodon* and *Trachelotractus*, and confirm their basal position within the Litostomatea. Our findings support a previous suggestion based on morphological features that the helicoprorodonids represent an independent group, possibly at the rank of order within the subclass Haptoria or even as a subclass within the class Litostomatea^95^,^96^,^97^. *Helicoprorodon* and *Trachelotractus* differ from other haptorids in having a peribuccal ridge with extrusomes, and specialized ciliary rows curving around the pharyngeal opening^98^.

### Further Insights into Phyllopharyngea and Nassophorea

Members of classes Phyllopharyngean and Nassophorean have a basket-like, ventrally opening oral apparatus or cyrtos^99^ as result of which it has long been assumed that these two groups are closely related^5^,^33^. The class Nassophorea sensu Lynn, 2008 comprises three orders, Synhymeniida, Nassulida and Microthoracida^5^. However, SSU rDNA-based phylogenies have shown that the order Synhymeniida clusters strongly with the class Phyllopharyngea rather than with the other two orders of Nassophorea. Based on these findings, Gong *et al.*^100^ revised the higher classification of these groups, regarding the synhymeniids as a subclass of the class Phyllopharyngea. In the present study, trees based on multi-gene data (Fig. 1A) also recover the synhymeniids as a distinct lineage within the Phyllopharyngea.

The group-name “Subkinetalia” was coined for phyllopharyngean super-clade comprising the subclasses Cyrtophoria, Chonotrichia, Rhynchodia and Suctoria, the synapomorphy of which is the possession of subkinetal microtubules^100^. The multi-gene based phylogeny in the present study is consistent with this finding^100^,^101^,^102^ and reveals a highly supported monophyletic Phyllopharyngea comprising two subgroups, the Subkinetalia (cyrtophorians, rhynchodians, chonotrichians, suctorians) and Synhymenia. Since the Subkinetalia represents a taxon between the ranks of subclass and class we believe it is more biological meaningful to refer to this as a superclade sharing same synapomorphic character.

Discotrichids are traditionally classified as a family within the nassophorean order Microthoracida^5^. However, Fan *et al.*^103^ established the order Discotrichida since discotrichids do not group with other microthoracids and are even distinct from all other nassophorean lineages. The present study also recovers the discotrichids (represented by *Discotricha*) as a distinct lineage that occupies a basal position within CONthreeP. However, evolutionary relationships between the discotrichids and other nassophoreans (microthoracids and nassulids) remain uncertain, probably due to undersampling within these groups.

The genus *Paranassula* in the nassophorean order Nassulida possesses distinct morphological characters that separates it from other nassulids, e.g. two polykinetids that are restricted to a shallow oral cavity and the presence of a paroral kinety^92^. Furthermore, phylogenetic analyses based on SSU rDNA and LSU rDNA sequence data suggest that *Paranassula* is related to Peniculia (class Oligohymeophorea)^102^. Our analyses with newly added gene sequence data support the most recent assignment for this taxon, i.e. *Paranassula* should be assigned to subclass Peniculia, class Oligohymenophorea. However, in contrast to the conclusion of Zhang *et al.*^102^ which suggested a resurrection of order Paranassulida Deroux in de Puytorac *et al.* (1993), we propose that *Paranassula* should be assigned to the order Peniculida, as a member of the family Paranassulidae Fauré-Fremiet, 1962. The phylogenetic positions of other genera in the family Paranassulidae, e.g. *Enneameron*, *Gullmarella*, remain uncertain due to undersampling.

### Systematically Ambiguous Taxa Find Their Close Relatives in the Class Plagiopylea

The systematic positions of the litostomatean genera *Askenasia*, *Cyclotrichium*, *Paraspathidium* and the prostome genus *Plagiocampa* have long been ambiguous. The former three have been variously assigned to the Cyclotrichida and Haptorida, or even as *incertae sedis* in the phylum Ciliophora^5^,^93^,^95^. Jankowski^104^ established the family Cyclotrichiidae and the order Cyclotrichida for *Cyclotrichium* which he assigned to the class Litostomatea, but failed to define these taxa. In the absence of any molecular data, Lynn^5^,^33^ regarded Cyclotrichiidae as a junior synonym of Didiniidae within the order Haptorida while retaining the order Cyclotrichida to include Mesodiniidae, e.g. *Askenasia*, *Mesodinium* and *Myrionecta*. Recent studies based on rDNA and the alpha-tubulin protein gene sequence data suggested that the two cyclotrichid genera *Askenasia*, *Cyclotrichium*, and the prostome genus *Paraspathidium*, should be removed from the class Litostomatea but failed to give their exact positions within the classes Plagiopylea and Prostomatea, respectively^94^,^102^. Lynn^5^ assigned the prostome genus *Plagiocampa* to the order Prorodontida. However, recent phylogenetic analyses recognized a distinct subclade comprising Plagiocampidae and two closely related genera *Urotricha* and *Cryptocaryon*^7^,^102^, which have a closer relationship to *Cyclotrichium* and *Paraspathidium* than to the prostomes^102^.

In the present study, based on multi-gene analyses, the concatenated gene trees robustly show that these four genera with uncertain taxonomic affiliations, i.e., *Askenasia*, *Cyclotrichium*, *Paraspathidium* and *Plagiocampa*, are most closely related to the plagiopyleans. Because of the highly specialized infraciliature and anaerobic life style of the plagiopyleans, their morphological synapomorphies are not well understood^5^. It is possible, for example, that a combination of the following features could define this clade (class Plagiopylea + *Askenasia* + *Paraspathidium*-*Cyclotrichium* + various prostomatean morphospecies represented by *Plagiocampa*): somatic monokinetids and an oralized somatic ciliature around a dominant cytostome consisting of densely ciliated dikinetids^105^,^106^,^107^. In addition, a brosse structure composed of dikinetids on the border of the oral slit has been commonly observed in *Paraspathidium*, *Urotricha* and *Plagiocampa*^106^,^107^. Based on the information above, we tentatively place *Askenasia Paraspathidium, Cyclotrichium* and various prostomatean morphospecies represented by *Plagiocampa* in the class Plagiopylea, pending further evidence including increased taxon sampling, ultrastructural studies and phylogenomic analyses.

### A Highly Supported Metaclade of Oligohymenophorea, Prostomatea and Plagiopylea within the CONthreeP

Several studies based on SSU rDNA sequence data have revealed a close evolutionary relationship among the classes Oligohymenophorea, Prostomatea and Plagiopylea^5^,^7^,^108^. Following the inclusion of sequences of additional genes and increased taxon sampling, the metaclade containing *Oligohymenophorea, Prostomatea and Plagiopylea* was recovered with high support. A close relationship between prostomes and oligohymenophoreans has been suggested based on similarities of their patterns of morphogenesis revealed by electron microscopy^102^,^109^,^110^,^111^. However, the phenotypic features that unite the riboclass Plagiopylea with Oligohymenophorea and Prostomatea remain unknown.

## Material and Methods

### DNA Extraction, Gene Sequencing, Dataset Assembly and Alignments

Gene sequence data were obtained for a total of 104 species representing almost all the main ciliate lineages. Genomic DNA extraction, PCR amplifications and sequencing were performed as described in previous studies^59^,^112^ for the following genes: completed sequence (~1800 bp) of the SSU rDNA; a partial sequence (~500 bp) of the ITS1-5.8S-ITS2; a partial sequence (~1800 bp) of the LSU rDNA; and, a partial sequence (~1000 bp) of the alpha-tubulin gene.

In total, 232 sequences were submitted to the GenBank database (Additional file 1: Table S1). In order to maximize the taxonomic diversity of ciliates included in our analyses, newly characterized sequences were combined with relevant sequences obtained from GenBank (Additional file 1: Table S2). Six datasets were evaluated: (1) concatenation of the aligned SSU rDNA, 5.8S DNA, LSU rDNA and alpha-tubulin amino acid sequences from datasets 2–5; (2) SSU rDNA sequences including all 152 group representatives; (3) 5.8S rDNA sequences of 113 taxa; (4) LSU rDNA sequences of 118 taxa; (5) alpha-tubulin amino acid sequences of 116 taxa; (6) alpha-tubulin nucleotide sequences, including the first two codon positions, of 116 taxa. Orthologs of alpha-tubulin for concatenations were selected according to Gao and Katz^15^.

Sequences were aligned using the GUIDANCE algorithm with default parameters in GUIDANCE web server^113^. Regions that could not be unambiguously aligned were excluded from the phylogenetic analyses. Because the ITS regions are too divergent to be aligned, only the 5.8S rDNA of the ITS1-5.8S-ITS2 region was used. The lengths of the final alignments of datasets (1)-(6) were 3794, 1661, 164, 1612, 357, 714 positions, respectively.

### Phylogenetic Analyses

Three apicomplexans and three dinoflagellates were used as outgroups (Additional file 1: Table S2)^44^. Maximum likelihood (ML) analyses were carried out using RAxML-HPC2 v7.6.6^114^ on CIPRES Science Gateway^115^. The DNA partition was analyzed with GTR + gamma. ProtTest 2.4^116^ selected the MtArt + I + G + F amino acid replacement matrix as the best-fitting model for alpha-tubulin amino acid sequences. The alpha-tubulin amino acid partition was run under the MtArt + gamma model as this was the best-fitting model available in RAxML. Support for the best-scoring ML tree came from 1000 bootstrap replicates. Bayesian inference (BI) analysis was performed with MrBayes 3.2.2^117^ on CIPRES Science Gateway using the GTR + I + G model for the DNA partition as selected by MrModeltest v.2.2^118^ and using mixed model for the alpha-tubulin amino acid partition. Markov chain Monte Carlo (MCMC) simulations were run with two sets of four chains for 4,000,000 generations with a sample frequency of 100 generations. The first 10,000 trees were discarded as burn-in. All remaining trees were used to calculate posterior probabilities using a majority rule consensus. Systematic schemes are mainly based on Lynn^5^ and Adl *et al.*^44^, except for some revisions made in the present study.

The approximately unbiased (AU) test^119^ was used to test the monophyly of the focal group against competing phylogenetic hypotheses (Table 1). Constrained ML trees enforcing the monophyly of the respective focal groups were generated based on SSU rDNA data. For all constraints, internal relationships within the constrained groups and among the remaining taxa were unspecified. The site-wise likelihoods for the resulting constrained topologies and the non-constrained ML topology were calculated using PAUP^120^ and were then analyzed in CONSEL^121^ with standard parameters to obtain *p*-values.

## Additional Information

**How to cite this article**: Gao, F. *et al.* The All-Data-Based Evolutionary Hypothesis of Ciliated Protists with a Revised Classification of the Phylum Ciliophora (Eukaryota, Alveolata). *Sci. Rep.* **6**, 24874; doi: 10.1038/srep24874 (2016).

## Supplementary Material

Supplementary Information

## Figures and Tables

**Table 1:** Approximately Unbiased test (AU) results based on SSU rDNA data.

Topology constraints	AU test
Climacostomidae	0.011
Urostylida + Pseudoamphisiellidae	0.007
Oligotrichia + *Lynnella*	0.682
Choreotrichia + *Lynnella*	0.397
Scuticociliatia	0.065
Philasterida + loxocephalids	0.028
Loxocephalida	0.021
Pleuronematida	7.00E-06
Peniculia	0.218
Peritrichia	0.657
Colpodea	0.365
Nassophorea	0.069
Cyclotrichida	0.088
Plagiopylea	0.695
Prostomatea	0.253

Notes: The topology constraints column refers to proposed taxonomic groups that were tested for monophyly through the approximately unbiased test (AU). Rejected monophyly (*p* < 0.05) is highlighted in gray.
