# Supplementary material for: The All-Data-Based Evolutionary Hypothesis of Ciliated Protists with a Revised Classification of the Phylum Ciliophora (Eukaryota, Alveolata)
Source: Sci Rep. 2016 Apr 29;6:24874. doi: 10.1038/srep24874 (PMC4850378; doi:10.1038/srep24874)

The All-Data-Based Evolutionary Hypothesis of Ciliated Protists with a Revised Classification of the Phylum Ciliophora (Eukaryota, Alveolata)

Feng Gao [a], Alan Warren [b], Qianqian Zhang [c], Jun Gong [c], Miao Miao [d], Ping Sun [e], Dapeng Xu [f], Jie Huang [g], Zhenzhen Yi [h,*] & Weibo Song [a,*]

[a] Institute of Evolution & Marine Biodiversity, Ocean University of China, Qingdao, China; [b] Department of Life Sciences, Natural History Museum, London, UK; [c] Yantai Institute of Coastal Zone Research, Chinese Academy of Sciences, Yantai, China; [d] College of Life Sciences, University of Chinese Academy of Sciences, Beijing, China; [e] Key Laboratory of the Ministry of Education for Coastal and Wetland Ecosystem, Xiamen University, Xiamen, China; [f] State Key Laboratory of Marine Environmental Science, Institute of Marine Microbes and Ecospheres, Xiamen University, Xiamen, China; [g] Institute of Hydrobiology, Chinese Academy of Sciences, Wuhan, China; [h] School of Life Science, South China Normal University, Guangzhou, China.

Running Head: Phylogeny and evolution of Ciliophora

* Address correspondence to Zhenzhen Yi, zyi@scnu.edu.cn; or Weibo Song, wsong@ouc.edu.cn

Table S1. List of species for which SSU rDNA, 5.8S rDNA, LSU rDNA, and alpha-tubulin were newly sequenced in the present work.

	Class	Subclass	Order	Family	Speicies	Sample sites	SSU rDNA	ITS1-5.8S-ITS2	LSU rDNA	a-tubulin
1	COLPODEA		Colpodida	Colpodidae	***Colpoda inflata***	A freshwater pond within the campus of the South China Normal University, Guangzhou (23° 09′N, 113° 22′ E)	KM222106	KM222071	KM222160	
2	HETEROTRICHEA		Heterotrichida	Climacostomidae	***Climacostomum virens***	No. 2 Bathing Beach, Qingdao (36° 03′ N, 120° 21′ E)			KM222165	KM221978
3	HETEROTRICHEA		Heterotrichida	Climacostomidae	***Fabrea salina***	Mariculture pond, Weifang (37°06″N, 119°30″)	KM222110	KM222078	KM222167	KM222005
4	HETEROTRICHEA		Heterotrichida	Condylostomatidae	***Condylostoma magnum***	Wanggezhuang mudflats, Qingdao (36° 17′ N, 120° 39′ E)	KM222108	KM222076	KM222163	KM221981
5	HETEROTRICHEA		Heterotrichida	Folliculinidae	***Folliculina simplex***	Zhongyuan Dock, Qingdao (36° 03′ N, 120° 19′ E)		KM222079	KM222168	KM221977
6	HETEROTRICHEA		Heterotrichida	Peritromidae	***Peritromus faurei***	Estuary of Baisha River, Qingdao (36° 15′ N, 120° 19′ E)		KM222080	KM222169	
7	HETEROTRICHEA		Heterotrichida	Spirostomidae	***Anigsteinia clarissima***	No. 1 Bathing Beach, Qingdao (36° 03′ N, 120° 21′ E)	KM222109	KM222077	KM222166	KM221982
8	HETEROTRICHEA		Heterotrichida	Spirostomidae	***Spirostomum* sp.**	A freshwater pond within the campus of the South China Normal University, Guangzhou (23° 09′N, 113° 22′ E)		KM222081	KM222170	KM222008
9	HETEROTRICHEA		Heterotrichida	Stentoridae	***Stentor* sp.**	Huguangyan scenic spot, Zhanjiang (21° 09′ N, 110° 18′ E)	KM222111	KM222082	KM222171	KM222003
10	KARYORELICTEA		Loxodida	Loxodidae	***Loxodes* cf. *striatus***	Seagull Island, Guagnzhou (22° 59′ N, 113° 32′ E)	KM222107	KM222075	KM222162	
11	KARYORELICTEA		Loxodida	Loxodidae	***Remanella granulosa***	Sculpture Park Bathing Beach, Qingdao (36° 05′N, 120° 27′ E)				KM221984
12	KARYORELICTEA		Protoheterotrichida	Geleiidae	***Geleia sinica***	No. 1 Bathing Beach, Qingdao (36° 03′ N, 120° 21′ E)		KM222074	KM222161	
13	KARYORELICTEA		Protostomatida	Kentrophoridae	***Kentrophoros gracilis***	No. 6 Bathing Beach, Qingdao (36° 03′ N, 120° 19′ E)				KM222029

77	LICNOPHORIEA		Licnophorida	Licnophoridae	***Licnophora* cf. *lyngbycola***	Daya Bay, Guangzhou (22° 36′N, 114° 33′ E)	FJ876955		KM222164	KM222007
14	LITOSTOMATEA	Haptoria	Haptorida	Acropisthiidae	***Chaenea* sp.**	Daya Bay, Guangzhou (22° 36′N, 114° 33′ E)		KM222059	KM222153	
15	LITOSTOMATEA	Haptoria	Haptorida	Helicoprorodontidae	***Helicoprorodon maximus***	Sculpture Park Bathing Beach, Qingdao (36° 05′N, 120° 27′ E)	KM222102	KM222061	KM222154	KM221989
16	LITOSTOMATEA	Haptoria	Haptorida	Tracheliidae	***Dileptus* sp.**	A freshwater pond within the campus of the South China Normal University, Guangzhou (23° 09′N, 113° 22′ E)	KM222100		KM222152	KM221975
17	LITOSTOMATEA	Haptoria	Pleurostomatida	Amphileptidae	***Amphileptus songi***	Circulating water system of a fish culture, Qingdao (36° 04′N, 120° 21′ E)	FJ876974	KM222058		
18	MESODINIEA		Mesodiniida	Mesodiniidae	***Mesodinium acarus***	Wanggezhuang mudflats, Qingdao (36° 17′ N, 120° 39′ E)	KM222101			
19	NASSOPHOREA		Discotrichida	Discotrichidae	***Discotricha papillifera***	Stone man bathing beach, Qingdao (36° 06′ N, 120° 29′ E)		KM222067		
20	NASSOPHOREA		Nassulida	Furgasoniidae	***Parafurgasonia* sp.**	上海 A freshwater pond in Changfeng Park, Shanghai, China (31° 14′ N, 121° 24′ E).		KM222070	KM222159	
21	NASSOPHOREA		Nassulida	Nassulidae	***Nassula labiata***	Daya Bay, Guangzhou (22° 36′N, 114° 33′ E)		KM222069		
22	OLIGOHYMENOPHOREA	Peniculia	Paranassulida	Paranassulidae	***Paranassula* sp.**	Daya Bay, Guangzhou (22° 36′N, 114° 33′ E)		KM222068		
23	OLIGOHYMENOPHOREA	Peniculia	Peniculida	Frontoniidae	***Frontonia canadensis***	Huguangyan scenic spot, Zhanjiang (21° 09′ N, 110° 18′ E)	KM222112	KM222083	KM222172	KM222002
24	OLIGOHYMENOPHOREA	Peniculia	Peniculida	Lembadionidae	***Lembadion* sp.**	Huguangyan scenic spot, Zhanjiang (21° 09′ N, 110° 18′ E)	KM222113	KM222084	KM222173	
25	OLIGOHYMENOPHOREA	Peritrichia	Sessilida	Epistylididae	***Epistylis* sp.**	Mangrove Reserve, Shenzhen (22° 32′N, 114° 00′ E)	KM222114	KM222085	KM222174	KM222025
26	OLIGOHYMENOPHOREA	Peritrichia	Sessilida	Epistylididae	***Pseudepistylis songi***	Nan'ao Island, Shantou (23° 28′N, 117° 06′ E)	KM222115	KM222086	KM222175	KM222023
27	OLIGOHYMENOPHOREA	Peritrichia	Sessilida	Opisthonectidae	***Opisthonecta* sp.**	Mangrove Reserve, Shenzhen (22° 32′N, 114° 00′ E)	KM222119	KM222090	KM222179	KM222024

28	OLIGOHYMENOPHOREA	Peritrichia	Sessilida	Vorticellidae	***Pseudovorticella shii***	Donghai Island, Zhanjiang (21°05′N, 110° 32′ E)	KM222116	KM222087	KM222176	KM222020
29	OLIGOHYMENOPHOREA	Peritrichia	Sessilida	Vorticellidae	***Vorticella chiangi***	Daya Bay, Guangzhou (22° 36′N, 114° 33′ E)	KM222117	KM222088	KM222177	KM222021
30	OLIGOHYMENOPHOREA	Peritrichia	Sessilida	Zoothamniidae	***Zoothamnium hentscheli***	Mipu Reserve, Hongkong (22° 30′N, 114° 04′ E)	KM222118	KM222089	KM222178	KM222022
31	OLIGOHYMENOPHOREA	Peritrichia	Sessilida	Zoothamniidae	***Zoothamnopsis sinica***	Seawater along the coast of Yellow Sea, Qingdao (36° 03′ N, 120° 22′ E)				KM222031
32	OLIGOHYMENOPHOREA	Scuticociliatia	Loxocephalida	Cinetochilidae	***Cinetochilum ovale***	A port in Qingdao (36° 05′ N, 120° 19′ E)				KM221956
33	OLIGOHYMENOPHOREA	Scuticociliatia	Loxocephalida	Cinetochilidae	***Pseudoplatynematum denticulatum***	No. 1 Bathing Beach, Qingdao (36° 03′ N, 120° 21′ E)				KM221967
34	OLIGOHYMENOPHOREA	Scuticociliatia	Loxocephalida	Cinetochilidae	***Sathrophilus holtae***	Stone man bathing beach, Qingdao (36° 06′ N, 120° 29′ E)				KM221968
35	OLIGOHYMENOPHOREA	Scuticociliatia	Loxocephalida	Loxocephalidae	***Cardiostomatella vermiformis***	Taiping Bay, Qingdao (36° 03′ N, 120° 22′ E)				KM221955
36	OLIGOHYMENOPHOREA	Scuticociliatia	Loxocephalida	Loxocephalidae	***Paratetrahymena wassi***	Daya Bay, Guangzhou (22° 36′N, 114° 33′ E)				KM221963
37	OLIGOHYMENOPHOREA	Scuticociliatia	Philasterida	Orchitophryidae	***Metanophrys sinensis***	Mariculture pond, Weifang (37°06″N, 119°30″)				KM221961
38	OLIGOHYMENOPHOREA	Scuticociliatia	Philasterida	Parauronematidae	***Miamiensis avidus***	Mariculture pond, Weifang (37°06″N, 119°30″)				KM221962
39	OLIGOHYMENOPHOREA	Scuticociliatia	Philasterida	Philasteridae	***Philasterides armatalis***	No. 6 Bathing Beach, Qingdao (36° 03′ N, 120° 19′ E)				KM221964
40	OLIGOHYMENOPHOREA	Scuticociliatia	Philasterida	Pseudocohnilembidae	***Pseudocohnilembus hargisi***	Nansan Island, Zhanjiang (21° 11′ N, 110° 27′ E)				KM221966
41	OLIGOHYMENOPHOREA	Scuticociliatia	Philasterida	Uronematidae	***Uronema marinum***	Seawater along the coast of Yellow Sea, Qingdao (36°18′ N, 120°43′ E)				KM221969
42	OLIGOHYMENOPHOREA	Scuticociliatia	Philasterida	Orchitophryidae	***Mesanophrys carcini***	Clear Water Bay, Hong Kong (22° 20′ N, 114°17′ E)				KM221960
43	OLIGOHYMENOPHOREA	Scuticociliatia	Pleuronematida	Ancistridae	***Ancistrum crassum***	From the mantle cavity of the marine mollusk *Ruditapes philippinarum*, Qingdao				KM221953

						(36° 04′ N, 120° 23′ E)				
44	OLIGOHYMENOPHOREA	Scuticociliatia	Pleuronematida	Ctedoctematidae	***Hippocomos salinus***	Yangkou sandy beach, Qingdao (36º14'N, 120º40'E)				KM221959
45	OLIGOHYMENOPHOREA	Scuticociliatia	Pleuronematida	Cyclidiidae	***Cristigera media***	No. 1 Bathing Beach, Qingdao (36° 03′ N, 120° 21′ E)				KM221957
46	OLIGOHYMENOPHOREA	Scuticociliatia	Pleuronematida	Cyclidiidae	***Protocyclidium citrullus***	Estuary of Baisha River, Qingdao (36° 15′ N, 120° 19′ E)				KM221958
47	OLIGOHYMENOPHOREA	Scuticociliatia	Pleuronematida	Eurystomateliidae	***Wilbertia typica***	Sculpture Park Bathing Beach, Qingdao (36° 05′N, 120° 27′ E)				KM221970
48	OLIGOHYMENOPHOREA	Scuticociliatia	Pleuronematida	Hemispeiridae	***Boveria subcyclindrica***	From the mantle cavity of the marine mollusk *Pinna pectinata*, Qingdao (36° 04′ N, 120° 23′ E)				KM221954
49	OLIGOHYMENOPHOREA	Scuticociliatia	Pleuronematida	Pleuronematidae	***Pleuronema setigerum***	A port in Qingdao (36° 05′ N, 120° 19′ E)				KM221965
50	PHYLLOPHARYNGEA	Cyrtophoria	Chlamydodontida	Chilodonellidae	***Trithigmostoma cucullulus***	Longxue Island, Guangzhou (22° 42′ N, 113° 39′ E)		KM222063	KM222156	KM221985
51	PHYLLOPHARYNGEA	Cyrtophoria	Chlamydodontida	Chlamydodontidae	***Chlamydodon mnemosyne***	Nansha Port, Guangzhou (22° 48′N, 113° 36′ E)				KM221986
52	PHYLLOPHARYNGEA	Cyrtophoria	Chlamydodontida	Lynchellidae	***Chlamydonella pseudochilodon***	Daya Bay, Guangzhou (22° 36′N, 114° 33′ E)				KM222001
53	PHYLLOPHARYNGEA	Cyrtophoria	Dysteriida	Dysteriidae	***Dysteria derouxi***	Mai Island, Qingdao (36° 04′ N, 120° 27′ E)	KM222105	KM222064		KM222030
54	PHYLLOPHARYNGEA	Rhynchodia	Hypocomatida	Hypocomidae	***Hypocoma acinetarum***	Zhongyuan Dock, Qingdao (36° 03′ N, 120° 19′ E)			KM222157	KM221983
55	PHYLLOPHARYNGEA	Suctoria	Exogenida	Ephelotidae	***Ephelota gemmipara***	Zhongyuan Dock, Qingdao (36° 03′ N, 120° 19′ E)		KM222065	KM222158	KM221976
56	PHYLLOPHARYNGEA	Synhymenia	Synhymeniida	Orthodonellidae	***Orthodonella* sp.**	Mai Island, Qingdao (36° 04′ N, 120° 27′ E)				KM221979
57	PHYLLOPHARYNGEA	Synhymenia	Synhymeniida	Orthodonellidae	***Zosterodasys agamalievi***	Daya Bay, Guangzhou (22° 36′N, 114° 33′ E)		KM222066		KM221995
58	PLAGIOPYLEA		Cyclotrichiida	Cyclotrichiida	***Paraspathidium apofuscum***	No. 6 Bathing Beach, Qingdao (36° 03′ N, 120° 19′ E)		KM222060		

59	PLAGIOPYLEA		Cyclotrichiida	Cyclotrichiidae	***Askenasia* sp.**	Gaoqiao, Zhanjiang (21° 37′ N, 109° 49′ E)				KM221994
60	PLAGIOPYLEA		Plagiopylida	Plagiopylidae	***Plagiopyla* sp.**	Mai Island, Qingdao (36° 04′ N, 120° 27′ E)		KM222073		
61	PLAGIOPYLEA		Plagiopylida	Sonderiidae	***Parasonderia vestita***	No. 6 Bathing Beach, Qingdao (36° 03′ N, 120° 19′ E)		KM222072		
62	PROSTOMATEA		Prorodontida	Colepidae	***Apocoleps* cf. *magnus***	Mai Island, Qingdao (36° 04′ N, 120° 27′ E)				KM221987
63	PROSTOMATEA		Prorodontida	Colepidae	***Nolandia orientalis***	Wanpingkou, Rizhao (35° 24′ N, 119° 34′ E)	KM222103	KM222062	KM222155	KM222006
64	PROSTOMATEA		Prorodontida	Colepidae	***Plagiopogon loricatus***	Sculpture Park Bathing Beach, Qingdao (36° 05′N, 120° 27′ E)				KM221990
65	PROSTOMATEA		Prorodontida	Placidae	***Placus salinus***	Mariculture pond, Weifang (37°06″N, 119°30″)				KM222004
66	PROSTOMATEA		Prorodontida	Prorodontidae	***Prorodon ovum***	No. 1 Bathing Beach, Qingdao (36° 03′ N, 120° 21′ E)	KM222104			KM221980
83	PROTOCRUZIEA		Protocruziida	Protocruziidae	***Protocruzia contrax***	Seawater along the coast of Yellow Sea, Qingdao (36°18′ N, 120°43′ E)		KM222050	KM222144	
67	SPIROTRICHEA	?	Lynnellida	Lynnelidae	***Lynnella semiglobulosa***	Daya Bay, Guangzhou (22° 36′N, 114° 33′ E)		KM222051	KM222145	KM221996
68	SPIROTRICHEA	Choreotrichia	Choreotrichida	Strombidinopsidae	***Strombidinopsis batos***	Mangrove Reserve, Shenzhen (22° 32′N, 114° 00′ E)	FJ881862	KM222054	KM222148	KM221998
69	SPIROTRICHEA	Choreotrichia	Tintinnida	Ptychocylididae	***Favella* cf. *campanula***	Daya Bay, Guangzhou (22° 36′N, 114° 33′ E)	KM222099	KM222057	KM222151	KM221992
70	SPIROTRICHEA	Hypotrichia	Discocephalida	Discocephalidae	***Discocephalus rotatorius***	Dameisha, Shenzhen (22° 36′N, 114° 19′ E)		KM222046	KM222138	
71	SPIROTRICHEA	Hypotrichia	Discocephalida	Discocephalidae	***Leptoamphisiella vermis***	Seawater along the coast of Yellow Sea, Qingdao (36° 03′ N, 120° 22′ E)		KM222044	KM222136	
85	SPIROTRICHEA	Hypotrichia	Discocephalida	Discocephalidae	***Pseudoamphisiella quadrinucleata***	Daya Bay, Guangzhou (22° 36′N, 114° 33′ E)			KM222123	
72	SPIROTRICHEA	Hypotrichia	Euplotida	Certesiidae	***Certesia quadrinucleta***	Taiping Bay, Qingdao (36° 03′ N, 120° 22′ E)	KM222097	KM222049	KM222143	KM221974
73	SPIROTRICHEA	Hypotrichia	Euplotida	Discocephalidae	***Paradiscocephalus***	A port in Qingdao (36° 05′ N, 120° 19′ E)		KM222047	KM222140	KM221988

					elongatus					
75	SPIROTRICHEA	Hypotrichia	Euplotida	Euplotidae	***Euplotes encysticus***	Daya Bay, Guangzhou (22° 36′N, 114° 33′ E)			KM222141	
74	SPIROTRICHEA	Hypotrichia	Euplotida	Euplotidae	***Euplotes sinicus***	Daya Bay, Guangzhou (22° 36′N, 114° 33′ E)			KM222139	
76	SPIROTRICHEA	Hypotrichia	Euplotida	Gastrocirrhidae	***Gastrocirrhus monilifer***	Zhongyuan Dock, Qingdao (36° 03′ N, 120° 19′ E)		KM222048	KM222142	
78	SPIROTRICHEA	Oligotrichia	Strombidiida	Strobilidiidae	***Pelagostrobilidium minutum***	Hengqin Island, Zhuhai (22° 10′ N, 113° 31′ E)		KM222055	KM222149	KM221991
79	SPIROTRICHEA	Oligotrichia	Strombidiida	Strobilidiidae	***Rimostrombidium veniliae***	Daya Bay, Guangzhou (22° 36′N, 114° 33′ E)		KM222056	KM222150	KM221993
80	SPIROTRICHEA	Oligotrichia	Strombidiida	Strombidiidae	***Spirostrombidium schizostomum***	Mangrove Reserve, Shenzhen (22° 32′N, 114° 00′ E)	KM222098	KM222053	KM222147	KM222000
81	SPIROTRICHEA	Oligotrichia	Strombidiida	Strombidiidae	***Strombidium stylifer***	Mangrove Reserve, Shenzhen (22° 32′N, 114° 00′ E)			KJ609058	KM221999
82	SPIROTRICHEA	Oligotrichia	Strombidiida	Tontoniidae	***Pseudotontonia simplicidens***	Daya Bay, Guangzhou (22° 36′N, 114° 33′ E)		KM222052	KM222146	KM221997
84	SPIROTRICHEA	Protohypotrichia	Kiitrichida	Kiitrichidae	***Caryotricha minuta***	Clear Water Bay, Hong Kong (22° 20′ N, 114° 17′ E)		KM222036	KM222125	KM222026
86	SPIROTRICHEA	Stichotrichia	Sporadotrichida	Oxytrichidae	***Hemigastrostyla enigmatica***	Clear Water Bay, Hong Kong (22° 20′ N, 114° 17′ E)	FJ870096	KM222035	KM222124	KM222027
87	SPIROTRICHEA	Stichotrichia	Sporadotrichida	Oxytrichidae	***Tachysoma pellionellum***	No. 1 Bathing Beach, Qingdao (36° 03′ N, 120° 21′ E)	KM222096	KM222045	KM222137	KM222015
88	SPIROTRICHEA	Stichotrichia	Sporadotrichida	Oxytrichidae	***Tetmemena puslata***	The intertidal zone at the estuary of the Pearl River, Seagull Village, Guangzhou (22° 59′ N, 113° 32′ E)	KM222092	KM222037	KM222126	KM222018
89	SPIROTRICHEA	Stichotrichia	Sporadotrichida	Trachelostylidae	***Trachelostyla pediculiformis***	Mangrove Reserve, Shenzhen (22° 32′N, 114° 00′ E)		KM222038	KM222127	KM222017
90	SPIROTRICHEA	Stichotrichia	Stichotrichida	Amphisiellidae	***Amphisiella annulata***	Zhongyuan Dock, Qingdao (36° 03′ N, 120° 19′ E)		KM222043	KM222135	KM222014
91	SPIROTRICHEA	Stichotrichia	Stichotrichida	Amphisiellidae	***Paracladotricha salina***	An offshore mollusc-farming pond of the Red island off the mouth area of Jiaozhou		KM222033	KM222121	KM222010

						Bay of Qingdao (N 36°04', E 120°18')				
92	SPIROTRICHEA	Stichotrichia	Stichotrichida	Amphisiellidae	***Uroleptoides qingdaoensis***	The lawn in University of Hebei, Baoding (38° 53′ N, 115° 31′ E)	KM222091	KM222032	KM222120	KM221971
93	SPIROTRICHEA	Stichotrichia	Stichotrichida	Kahliellidae	***Pseudokahliella marina***	Daya Bay, Guangzhou (22° 36′N, 114° 33′ E)	KM222095	KM222041	KM222133	KM222019
94	SPIROTRICHEA	Stichotrichia	Stichotrichida	Spirofilidae	***Strongylidium orie ntale***	Mipu Reserve, Hongkong (22° 30′N, 114° 04′ E)		KM222034	KM222122	KM221972
95	SPIROTRICHEA	Stichotrichia	Urostylida	Epiclintidae	***Epiclintes auricularis auricularis***	Mai Island, Qingdao (36° 04′ N, 120° 27′ E)			KM222128	
96	SPIROTRICHEA	Stichotrichia	Urostylida	Pseudokeronopsidae	***Nothoholosticha fasciola***	Mai Island, Qingdao (36° 04′ N, 120° 27′ E)				KM222011
97	SPIROTRICHEA	Stichotrichia	Urostylida	Pseudokeronopsidae	***Pseudokeronopsis flava***	Coastal seawater, Zhanjiang (21°15′ N, 110° 22′ E)				KM221973
98	SPIROTRICHEA	Stichotrichia	Urostylida	Pseudokeronopsidae	***Uroleptopsis citrina***	No. 2 Bathing Beach, Qingdao (36° 03′ N, 120° 21′ E)				KM222013
99	SPIROTRICHEA	Stichotrichia	Urostylida	Pseudourostylidae	***Pseudourostyla* sp.**	Qi'ao Island, Zhuhai (22° 26′ N, 113° 38′ E)		KM222042	KM222134	KM222016
100	SPIROTRICHEA	Stichotrichia	Urostylida	Urostylidae	***Anteholosticha petzi***	Estuary of Haibo River, Qingdao (36° 07′ N, 120° 20′ E)	KM222093	KM222039	KM222130	
101	SPIROTRICHEA	Stichotrichia	Urostylida	Urostylidae	***Holosticha diademata***	Seawater along the coast of Yellow Sea, Qingdao (36° 03′ N, 120° 22′ E)			KM222131	KM222009
102	SPIROTRICHEA	Stichotrichia	Urostylida	Urostylidae	***Holosticha heterofoissneri***	No. 6 Bathing Beach, Qingdao (36° 03′ N, 120° 19′ E)	KM222094	KM222040	KM222132	KM222012
103	SPIROTRICHEA	Stichotrichia	Urostylida	Urostylidae	***Neourostylopsis flavicana***	Mangrove Reserve, Shenzhen (22° 32′N, 114° 00′ E)				KM222028
104	SPIROTRICHEA	Stichotrichia	Urostylida	Urostylidae	***Parabirojimia multinucleata***	Daya Bay, Guangzhou (22° 36′N, 114° 33′ E)			KM222129	

Table S2. List of species for which SSU rDNA, 5.8S rDNA, LSU rDNA, and alpha-tubulin were used in the present work.

	Class	Subclass	Order	Family	Taxon	Source	18S	ITS1-5.8S-ITS2	28S	a-tubulin
1	ARMOPHOREA		Armophorida	Metopidae	***Metopus palaeformis***	GenBank	AY007450	NA	NA	AY041133
2	ARMOPHOREA		Clevelandellida	Clevelandellidae	***Clevelandella panesthiae***	GenBank	KC139719	KC460347	NA	NA
3	ARMOPHOREA		Clevelandellida	Nyctotheridae	***Nyctotherus ovalis***	GenBank	AJ222678	AJ006714	NA	DQ665926
4	COLPODEA		Bryometopida	Bryometopida	***Bryometopus atypicus***	GenBank	EU039886	NA	NA	NA
5	COLPODEA		Bryophryida	Bryophryidae	***Notoxoma parabryophryides***	GenBank	EU039903	NA	NA	NA
6	COLPODEA		Bursariomorphida	Bursariidae	***Bursaria truncatella***	GenBank	U82204	NA	NA	NA
7	COLPODEA		Colpodida	Colpodidae	***Colpoda inflata***	Lab of Protozoology	KM222106	KM222071	KM222160	NA
8	COLPODEA		Colpodida	Hausmanniellidae	***Bresslauides discoideus***	GenBank	HM140394	NA	HM122014	NA
9	COLPODEA		Cyrtolophosidida	Platyophryidae	***Platyophryides magnus***	GenBank	HM140395	NA	HM122030	NA
10	COLPODEA		Sorogenida	Sorogenidae	***Sorogena stoianovitchae***	GenBank	AF300285	NA	NA	NA
11	HETEROTRICHEA		Heterotrichida	Climacostomidae	***Climacostomum virens***	Lab of Protozoology	EU583990	NA	KM222165	KM221978
12	HETEROTRICHEA		Heterotrichida	Climacostomidae	***Fabrea salina***	Lab of Protozoology	KM222110	KM222078	KM222167	KM222005
13	HETEROTRICHEA		Heterotrichida	Condylostomatidae	***Condylostoma magnum***	Lab of Protozoology	KM222108	KM222076	KM222163	KM221981
14	HETEROTRICHEA		Heterotrichida	Folliculinidae	***Folliculina simplex***	Lab of Protozoology	EU583992	KM222079	KM222168	KM221977
15	HETEROTRICHEA		Heterotrichida	Peritromidae	***Peritromus faurei***	Lab of Protozoology	EU583993	KM222080	KM222169	NA
16	HETEROTRICHEA		Heterotrichida	Spirostomidae	***Anigsteinia clarissima***	Lab of Protozoology	KM222109	KM222077	KM222166	KM221982
17	HETEROTRICHEA		Heterotrichida	Spirostomidae	***Spirostomum* sp.**	Lab of Protozoology	FJ998027	KM222081	KM222170	KM222008
18	HETEROTRICHEA		Heterotrichida	Stentoridae	***Stentor* sp.**	Lab of Protozoology	KM222111	KM222082	KM222171	KM222003
19	KARYORELICTEA		?	Wilbertomorphidae	***Wilbertomorpha colpoda***	Lab of Protozoology	KC461119	NA	NA	NA
20	KARYORELICTEA		Loxodida	Loxodidae	***Loxodes* cf. *striatus***	Lab of Protozoology	KM222107	KM222075	KM222162	NA
21	KARYORELICTEA		Loxodida	Loxodidae	***Remanella granulosa***	Lab of Protozoology	JQ768408	NA	NA	KM221984
22	KARYORELICTEA		Protoheterotrichida	Geleiidae	***Geleia sinica***	Lab of Protozoology	JF437558	KM222074	KM222161	NA
23	KARYORELICTEA		Protostomatida	Kentrophoridae	***Kentrophoros gracilis***	Lab of Protozoology	FJ467506	NA	NA	KM222029
24	KARYORELICTEA		Protostomatida	Trachelocercidae	***Trachelocerca sagitta***	Lab of Protozoology	KC542935	NA	NA	NA

25	LICNOPHORIEA		Licnophorida	Licnophoridae	***Licnophora* cf. *lyngbycola***	Lab of Protozoology	FJ876955	NA	KM222164	KM222007
26	LITOSTOMATEA	Haptoria	Haptorida	Acropisthiidae	***Chaenea* sp.**	Lab of Protozoology	FJ876970	KM222059	KM222153	JF975407
27	LITOSTOMATEA	Haptoria	Haptorida	Helicoprorodontidae	***Helicoprorodon maximus***	Lab of Protozoology	KM222102	KM222061	KM222154	KM221989
28	LITOSTOMATEA	Haptoria	Haptorida	Helicoprorodontidae	***Trachelotractus entzi***	Lab of Protozoology	FJ463745	NA	JF975394	JF975403
29	LITOSTOMATEA	Haptoria	Haptorida	Lacrymariidae	***Phialina salinarum***	Lab of Protozoology	EU242508	NA	JF975395	JF975404
30	LITOSTOMATEA	Haptoria	Haptorida	Tracheliidae	***Dileptus* sp.**	Lab of Protozoology	KM222100	NA	KM222152	KM221975
31	LITOSTOMATEA	Haptoria	Pleurostomatida	Amphileptidae	***Amphileptus songi***	Lab of Protozoology	FJ876974	KM222058	JF975389	JF975397
32	LITOSTOMATEA	Haptoria	Pleurostomatida	Amphileptidae	***Epiphyllum shenzhenense***	Lab of Protozoology	GU574809	NA	JF975392	JF975408
33	LITOSTOMATEA	Trichostomatia	Entodiniomorphida	Troglodytellidae	***Troglodytella abrassarti***	GenBank	AB437346	EU680311	NA	NA
34	LITOSTOMATEA	Trichostomatia	Vestibuliferida	Balantidiidae	***Balantidium entozoon***	GenBank	EU581716	JQ408694	NA	NA
35	MESODINIEA		MESODINIIDA	Mesodiniidae	***Mesodinium acarus***	Lab of Protozoology	KM222101	NA	NA	NA
36	NASSOPHOREA		Discotrichida	Discotrichidae	***Discotricha papillifera***	Lab of Protozoology	JQ918368	KM222067	KC832966	NA
37	NASSOPHOREA		Microthoracida	Leptopharyngidae	***Leptopharynx costatus***	Lab of Protozoology	EU286811	NA	KC832958	NA
38	NASSOPHOREA		Nassulida	Furgasoniidae	***Parafurgasonia* sp.**	Lab of Protozoology	KC832955	KM222070	KM222159	NA
39	NASSOPHOREA		Nassulida	Nassulidae	***Nassula labiata***	Lab of Protozoology	KC832949	KM222069	KC832957	NA
40	OLIGOHYMENOPHOREA	Apostomatia	Apostomatida	Colliniidae	***Pseudocollinia beringensis***	GenBank	HQ591474	HQ591474	HQ591474	NA
41	OLIGOHYMENOPHOREA	Astomatia	Astomatida	Anoplophryidae	***Almophrya bivacuolata***	GenBank	HQ446281	HQ446281	NA	NA
42	OLIGOHYMENOPHOREA	Hymenostomatia	Ophryoglenida	Ichthyophthiriidae	***Ichthyophthirius multifiliis***	GenBank	U17354	DQ270016	EU185635	EGR31414
43	OLIGOHYMENOPHOREA	Hymenostomatia	Tetrahymenida	Tetrahymenidae	***Tetrahymena thermophila***	GenBank	X56165	JN547815	JN547815	AAA21350
44	OLIGOHYMENOPHOREA	Peniculia	Paranassulida	Paranassulidae	***Paranassula* sp.**	Lab of Protozoology	KC832956	KM222068	KC832964	NA
45	OLIGOHYMENOPHOREA	Peniculia	Peniculida	Frontoniidae	***Frontonia canadensis***	Lab of Protozoology	KM222112	KM222083	KM222172	KM222002
46	OLIGOHYMENOPHOREA	Peniculia	Peniculida	Lembadionidae	***Lembadion* sp.**	Lab of Protozoology	KM222113	KM222084	KM222173	NA
47	OLIGOHYMENOPHOREA	Peniculia	Peniculida	Parameciidae	***Paramecium tetraurelia***	GenBank	AY102613	JX661316	EU828456	CAA67847
48	OLIGOHYMENOPHOREA	Peniculia	Urocentrida	Urocentridae	***Urocentrum turbo***	GenBank	AF255357	EF114295	KF287650	NA
49	OLIGOHYMENOPHOREA	Peritrichia	Mobilida	Trichodinidae	***Trichodina heterodentata***	GenBank	AY788099	NA	NA	EF569680
50	OLIGOHYMENOPHOREA	Peritrichia	Mobilida	Trichodinidae	***Trichodinella myakkae***	GenBank	AY102176	NA	NA	EF569678
51	OLIGOHYMENOPHOREA	Peritrichia	Sessilida	Epistylididae	***Epistylis* sp.**	Lab of Protozoology	KM222114	KM222085	KM222174	KM222025

52	OLIGOHYMENOPHOREA	Peritrichia	Sessilida	Epistylididae	***Pseudepistylis songi***	Lab of Protozoology	KM222115	KM222086	KM222175	KM222023
53	OLIGOHYMENOPHOREA	Peritrichia	Sessilida	Opisthonectidae	***Opisthonecta* sp.**	Lab of Protozoology	KM222119	KM222090	KM222179	KM222024
54	OLIGOHYMENOPHOREA	Peritrichia	Sessilida	Vorticellidae	***Carchesium polypinum***	GenBank	GU987027	GU987034	NA	FJ883553
55	OLIGOHYMENOPHOREA	Peritrichia	Sessilida	Vorticellidae	***Epicarchesium abrae***	GenBank	DQ190462	EU340854	NA	NA
56	OLIGOHYMENOPHOREA	Peritrichia	Sessilida	Vorticellidae	***Pseudovorticella shii***	Lab of Protozoology	KM222116	KM222087	KM222176	KM222020
57	OLIGOHYMENOPHOREA	Peritrichia	Sessilida	Vorticellidae	***Vorticella chiangi***	Lab of Protozoology	KM222117	KM222088	KM222177	KM222021
58	OLIGOHYMENOPHOREA	Peritrichia	Sessilida	Zoothamniidae	***Zoothamnium hentscheli***	Lab of Protozoology	KM222118	KM222089	KM222178	KM222022
59	OLIGOHYMENOPHOREA	Peritrichia	Sessilida	Zoothamniidae	***Zoothamnopsis sinica***	Lab of Protozoology	DQ190469	NA	NA	KM222031
60	OLIGOHYMENOPHOREA	Scuticociliatia	Loxocephalida	Cinetochilidae	***Cinetochilum ovale***	Lab of Protozoology	FJ870103	JX310025	JX310025	KM221956
61	OLIGOHYMENOPHOREA	Scuticociliatia	Loxocephalida	Cinetochilidae	***Pseudoplatynematum denticulatum***	Lab of Protozoology	JX310020	JX310004	JX310028	KM221967
62	OLIGOHYMENOPHOREA	Scuticociliatia	Loxocephalida	Cinetochilidae	***Sathrophilus holtae***	Lab of Protozoology	FJ868188	JX310029	JX310029	KM221968
63	OLIGOHYMENOPHOREA	Scuticociliatia	Loxocephalida	Loxocephalidae	***Cardiostomatella vermiformis***	Lab of Protozoology	AY881632	JX310024	JX310024	KM221955
64	OLIGOHYMENOPHOREA	Scuticociliatia	Loxocephalida	Loxocephalidae	***Paratetrahymena wassi***	Lab of Protozoology	GQ292767	JX310009	JX310027	KM221963
65	OLIGOHYMENOPHOREA	Scuticociliatia	Philasterida	Orchitophryidae	***Metanophrys sinensis***	Lab of Protozoology	HM236336	JN885092	JN885114	KM221961
66	OLIGOHYMENOPHOREA	Scuticociliatia	Philasterida	Parauronematidae	***Miamiensis avidus***	Lab of Protozoology	JN885091	JN885095	JN885115	KM221962
67	OLIGOHYMENOPHOREA	Scuticociliatia	Philasterida	Philasteridae	***Philasterides armatalis***	Lab of Protozoology	FJ848877	JN885098	JN885120	KM221964
68	OLIGOHYMENOPHOREA	Scuticociliatia	Philasterida	Pseudocohnilembidae	***Pseudocohnilembus hargisi***	Lab of Protozoology	JN885090	JN885100	JN885122	KM221966
69	OLIGOHYMENOPHOREA	Scuticociliatia	Philasterida	Uronematidae	***Uronema marinum***	Lab of Protozoology	GQ465466	JN885102	JN885124	KM221969
70	OLIGOHYMENOPHOREA	Scuticociliatia	Philasterida	Orchitophryidae	***Mesanophrys carcini***	Lab of Protozoology	JN885086	JN885104	JN885113	KM221960
71	OLIGOHYMENOPHOREA	Scuticociliatia	Pleuronematida	Ancistridae	***Ancistrum crassum***	Lab of Protozoology	HM236340	KF256831	KF256825	KM221953
72	OLIGOHYMENOPHOREA	Scuticociliatia	Pleuronematida	Ctedoctematidae	***Hippocomos salinus***	Lab of Protozoology	JX310012	JX310023	JX310023	KM221959
73	OLIGOHYMENOPHOREA	Scuticociliatia	Pleuronematida	Cyclidiidae	***Cristigera media***	Lab of Protozoology	FJ868180	KF256827	KF256827	KM221957
74	OLIGOHYMENOPHOREA	Scuticociliatia	Pleuronematida	Cyclidiidae	***Protocyclidium citrullus***	Lab of Protozoology	KF256820	KF256832	KF256823	KM221958
75	OLIGOHYMENOPHOREA	Scuticociliatia	Pleuronematida	Eurystomateliidae	***Wilbertia typica***	Lab of Protozoology	JX310022	JX310031	JX310031	KM221970
76	OLIGOHYMENOPHOREA	Scuticociliatia	Pleuronematida	Hemispeiridae	***Boveria subcyclindrica***	Lab of Protozoology	FJ848878	NA	KF256826	KM221954
77	OLIGOHYMENOPHOREA	Scuticociliatia	Pleuronematida	Pleuronematidae	***Pleuronema setigerum***	Lab of Protozoology	FJ848874	JX310006	NA	KM221965
78	PHYLLOPHARYNGEA	Chonotrichia	Cryptogemmida	Isochonidae	***Isochona* sp.**	GenBank	AY242116	NA	NA	NA

79	PHYLLOPHARYNGEA	Cyrtophoria	Chlamydodontida	Chilodonellidae	***Trithigmostoma cucullulus***	Lab of Protozoology	FJ998037	KM222063	KM222156	KM221985
80	PHYLLOPHARYNGEA	Cyrtophoria	Chlamydodontida	Chlamydodontidae	***Chlamydodon mnemosyne***	Lab of Protozoology	FJ998031	NA	NA	KM221986
81	PHYLLOPHARYNGEA	Cyrtophoria	Chlamydodontida	Lynchellidae	***Chlamydonella pseudochilodon***	Lab of Protozoology	FJ998032	NA	NA	KM222001
82	PHYLLOPHARYNGEA	Cyrtophoria	Dysteriida	Dysteriidae	***Dysteria derouxi***	Lab of Protozoology	KM222105	KM222064	KC832960	KM222030
83	PHYLLOPHARYNGEA	Rhynchodia	Hypocomatida	Hypocomidae	***Hypocoma acinetarum***	Lab of Protozoology	JN867019	NA	KM222157	KM221983
84	PHYLLOPHARYNGEA	Suctoria	Endogenida	Acinetidae	***Acineta tuberosa***	Lab of Protozoology	FJ865206	NA	NA	NA
85	PHYLLOPHARYNGEA	Suctoria	Evaginogenida	Heliophryidae	***Heliophrya erhardi***	GenBank	AY007446	NA	NA	AY041138
86	PHYLLOPHARYNGEA	Suctoria	Exogenida	Ephelotidae	***Ephelota gemmipara***	Lab of Protozoology	EU600180	KM222065	KM222158	KM221976
87	PHYLLOPHARYNGEA	Suctoria	Exogenida	Paracinetidae	***Paracineta limbata***	Lab of Protozoology	FJ865207	NA	NA	NA
88	PHYLLOPHARYNGEA	Synhymenia	Synhymeniida	Orthodonellidae	***Orthodonella* sp.**	Lab of Protozoology	KC832952	NA	KC832963	KM221979
89	PHYLLOPHARYNGEA	Synhymenia	Synhymeniida	Orthodonellidae	***Zosterodasys agamalievi***	Lab of Protozoology	FJ998040	KM222066	KC832961	KM221995
90	PLAGIOPYLEA		Cyclotrichiida	Cyclotrichiidae	***Paraspathidium apofuscum***	Lab of Protozoology	FJ875140	KM222060	JF975391	JF975401
91	PLAGIOPYLEA		Cyclotrichiida	Cyclotrichiidae	***Askenasia* sp.**	Lab of Protozoology	KC771341	KC771343	KC771338	KM221994
92	PLAGIOPYLEA		Cyclotrichiida	Cyclotrichiidae	***Cyclotrichium cyclokaryon***	Lab of Protozoology	FJ876971	NA	JF975396	JF975406
93	PLAGIOPYLEA		Cyclotrichiida	Plagiocampidae	***Plagiocampa sp.***	Lab of Protozoology	KC832950	KC771347	KC832962	NA
94	PLAGIOPYLEA		Odontostomatida	Epalxellidae	***Epalxella antiquorum***	GenBank	EF014286	NA	NA	NA
95	PLAGIOPYLEA		Plagiopylida	Plagiopylidae	***Plagiopyla* sp.**	Lab of Protozoology	FJ875150	KM222073	NA	JF975402
96	PLAGIOPYLEA		Plagiopylida	Sonderiidae	***Parasonderia vestita***	Lab of Protozoology	JN857941	KM222072	NA	NA
97	PROSTOMATEA		Prorodontida	Colepidae	***Apocoleps* cf. *magnus***	Lab of Protozoology	HM747137	NA	NA	KM221987
98	PROSTOMATEA		Prorodontida	Colepidae	***Nolandia orientalis***	Lab of Protozoology	KM222103	KM222062	KM222155	KM222006
99	PROSTOMATEA		Prorodontida	Colepidae	***Plagiopogon loricatus***	Lab of Protozoology	KC771342	KC771344	KC771339	KM221990
100	PROSTOMATEA		Prorodontida	Placidae	***Placus salinus***	Lab of Protozoology	KC832954	KC771346	KC832959	KM222004
101	PROSTOMATEA		Prorodontida	Prorodontidae	***Prorodon ovum***	Lab of Protozoology	KM222104	KC771345	KC771340	KM221980
102	PROTOCRUZIEA		Protocruziida	Protocruziidae	***Protocruzia contrax***	Lab of Protozoology	DQ190467	KM222050	KM222144	EF198665
103	SPIROTRICHEA	?	Lynnellida	Lynnelidae	***Lynnella semiglobulosa***	Lab of Protozoology	FJ876965	KM222051	KM222145	KM221996
104	SPIROTRICHEA	Choreotrichia	Choreotrichida	Strombidinopsidae	***Strombidinopsis batos***	Lab of Protozoology	FJ881862	KM222054	KM222148	KM221998
105	SPIROTRICHEA	Choreotrichia	Tintinnida	Codonellidae	***Tintinnopsis tubulosoides***	GenBank	AF399111	AF399030	NA	AF399112
106	SPIROTRICHEA	Choreotrichia	Tintinnida	Metacylididae	***Helicostomella subulata***	GenBank	JQ716991	JX000468	JN831876	NA

107	SPIROTRICHEA	Choreotrichia	Tintinnida	Ptychocylididae	***Favella* cf. *campanula***	Lab of Protozoology	KM222099	KM222057	KM222151	KM221992
108	SPIROTRICHEA	Hypotrichia	Discocephalida	Discocephalidae	***Discocephalus rotatorius***	Lab of Protozoology	JX460983	KM222046	KM222138	JQ736719
109	SPIROTRICHEA	Hypotrichia	Discocephalida	Discocephalidae	***Leptoamphisiella vermis***	Lab of Protozoology	FJ865203	KM222044	KM222136	JQ736707
110	SPIROTRICHEA	Hypotrichia	Discocephalida	Discocephalidae	***Paradiscocephalus elongatus***	Lab of Protozoology	EU684746	KM222047	KM222140	KM221988
111	SPIROTRICHEA	Hypotrichia	Discocephalida	Discocephalidae	***Pseudoamphisiella quadrinucleata***	Lab of Protozoology	EU518416	GQ246484	KM222123	GQ258109
112	SPIROTRICHEA	Hypotrichia	Euplotida	Aspidiscidae	***Aspidisca leptaspis***	Lab of Protozoology	EU880597	FJ600369	JF694064	JQ736687
113	SPIROTRICHEA	Hypotrichia	Euplotida	Certesiidae	***Certesia quadrinucleta***	Lab of Protozoology	KM222097	KM222049	KM222143	KM221974
114	SPIROTRICHEA	Hypotrichia	Euplotida	Euplotidae	***Euplotes encysticus***	Lab of Protozoology	FJ346569	NA	KM222141	JQ736725
115	SPIROTRICHEA	Hypotrichia	Euplotida	Euplotidae	***Euplotes sinicus***	Lab of Protozoology	FJ876980	NA	KM222139	JQ736734
116	SPIROTRICHEA	Hypotrichia	Euplotida	Gastrocirrhidae	***Gastrocirrhus monilifer***	Lab of Protozoology	DQ864734	KM222048	KM222142	JQ918351
117	SPIROTRICHEA	Hypotrichia	Euplotida	Uronychiidae	***Diophrys parappendiculata***	Lab of Protozoology	EU267928	JF694074	JF694056	JQ736739
118	SPIROTRICHEA	Hypotrichia	Euplotida	Uronychiidae	***Uronychia sinica***	Lab of Protozoology	FJ876982	JF694083	JF694063	JQ736736
119	SPIROTRICHEA	Oligotrichia	Strombidiida	Strobilidiidae	***Pelagostrobilidium minutum***	Lab of Protozoology	FJ876959	KM222055	KM222149	KM221991
120	SPIROTRICHEA	Oligotrichia	Strombidiida	Strobilidiidae	***Rimostrombidium veniliae***	Lab of Protozoology	FJ876964	KM222056	KM222150	KM221993
121	SPIROTRICHEA	Oligotrichia	Strombidiida	Strombidiidae	***Spirostrombidium schizostomum***	Lab of Protozoology	KM222098	KM222053	KM222147	KM222000
122	SPIROTRICHEA	Oligotrichia	Strombidiida	Strombidiidae	***Strombidium stylifer***	Lab of Protozoology	JX012185	JN853794	KJ609058	KM221999
123	SPIROTRICHEA	Oligotrichia	Strombidiida	Tontoniidae	***Pseudotontonia simplicidens***	Lab of Protozoology	FJ422993	KM222052	KM222146	KM221997
124	SPIROTRICHEA	Phacodiniidia	Phacodiniida	Phacodiniidae	***Phacodinium metchnikoffi***	GenBank	AJ277877	NA	HM122023	AY554040
125	SPIROTRICHEA	Protohypotrichia	Kiitrichida	Kiitrichidae	***Caryotricha minuta***	Lab of Protozoology	EU275202	KM222036	KM222125	KM222026
126	SPIROTRICHEA	Protohypotrichia	Kiitrichida	Kiitrichidae	***Kiitrichia marina***	Lab of Protozoology	AY896768	NA	NA	NA
127	SPIROTRICHEA	Stichotrichia	Sporadotrichida	Halteriidae	***Halteria grandinella***	GenBank	AF508759	AF508759	AF508759	AY041120
128	SPIROTRICHEA	Stichotrichia	Sporadotrichida	Halteriidae	***Meseres corlissi***	GenBank	EU399527	EU399527	EU399527	NA
129	SPIROTRICHEA	Stichotrichia	Sporadotrichida	Oxytrichidae	***Hemigastrostyla enigmatica***	Lab of Protozoology	FJ870096	KM222035	KM222124	KM222027
130	SPIROTRICHEA	Stichotrichia	Sporadotrichida	Oxytrichidae	***Oxytricha granulifera***	GenBank	AF508762	AF508762	AF508762	Z11763
131	SPIROTRICHEA	Stichotrichia	Sporadotrichida	Oxytrichidae	***Tachysoma pellionellum***	Lab of Protozoology	KM222096	KM222045	KM222137	KM222015
132	SPIROTRICHEA	Stichotrichia	Sporadotrichida	Oxytrichidae	***Tetmemena puslata***	Lab of Protozoology	KM222092	KM222037	KM222126	KM222018

133	SPIROTRICHEA	Stichotrichia	Sporadotrichida	Trachelostylidae	***Trachelostyla pediculiformis***	Lab of Protozoology	DQ057346	KM222038	KM222127	KM222017
134	SPIROTRICHEA	Stichotrichia	Stichotrichida	Amphisiellidae	***Amphisiella annulata***	Lab of Protozoology	DQ832260	KM222043	KM222135	KM222014
135	SPIROTRICHEA	Stichotrichia	Stichotrichida	Amphisiellidae	***Paracladotricha salina***	Lab of Protozoology	FJ870085	KM222033	KM222121	KM222010
136	SPIROTRICHEA	Stichotrichia	Stichotrichida	Amphisiellidae	***Uroleptoides qingdaoensis***	Lab of Protozoology	KM222091	KM222032	KM222120	KM221971
137	SPIROTRICHEA	Stichotrichia	Stichotrichida	Kahliellidae	***Pseudokahliella marina***	Lab of Protozoology	KM222095	KM222041	KM222133	KM222019
138	SPIROTRICHEA	Stichotrichia	Stichotrichida	Plagiotomidae	***Plagiotoma lumbrici***	GenBank	AY547545	NA	NA	NA
139	SPIROTRICHEA	Stichotrichia	Stichotrichida	Spirofilidae	***Strongylidium orientale***	Lab of Protozoology	KC153532	KM222034	KM222122	KM221972
140	SPIROTRICHEA	Stichotrichia	Urostylida	Bergeriellidae	***Bergeriella ovata***	Lab of Protozoology	FJ754026	GQ246479	JQ424856	GQ258113
141	SPIROTRICHEA	Stichotrichia	Urostylida	Epiclintidae	***Epiclintes auricularis auricularis***	Lab of Protozoology	FJ008721	GQ246480	KM222128	GQ262001
142	SPIROTRICHEA	Stichotrichia	Urostylida	Pseudokeronopsidae	***Nothoholosticha fasciola***	Lab of Protozoology	FJ377548	FJ858212	JQ424846	KM222011
143	SPIROTRICHEA	Stichotrichia	Urostylida	Pseudokeronopsidae	***Pseudokeronopsis flava***	Lab of Protozoology	DQ227798	DQ503579	JQ424835	KM221973
144	SPIROTRICHEA	Stichotrichia	Urostylida	Pseudokeronopsidae	***Thigmokeronopsis stoecki***	Lab of Protozoology	EU220226	GQ246485	JQ424844	EU678915
145	SPIROTRICHEA	Stichotrichia	Urostylida	Pseudokeronopsidae	***Uroleptopsis citrina***	Lab of Protozoology	FJ870094	JQ424858	JQ424850	KM222013
146	SPIROTRICHEA	Stichotrichia	Urostylida	Pseudourostylidae	***Pseudourostyla* sp.**	Lab of Protozoology	FJ775725	KM222042	KM222134	KM222016
147	SPIROTRICHEA	Stichotrichia	Urostylida	Urostylidae	***Anteholosticha petzi***	Lab of Protozoology	KM222093	KM222039	KM222130	GQ258105
148	SPIROTRICHEA	Stichotrichia	Urostylida	Urostylidae	***Apokeronopsis bergeri***	Lab of Protozoology	DQ777742	DQ777741	JQ424840	GQ258112
149	SPIROTRICHEA	Stichotrichia	Urostylida	Urostylidae	***Holosticha diademata***	Lab of Protozoology	DQ059583	EU925647	KM222131	KM222009
150	SPIROTRICHEA	Stichotrichia	Urostylida	Urostylidae	***Holosticha heterofoissneri***	Lab of Protozoology	KM222094	KM222040	KM222132	KM222012
151	SPIROTRICHEA	Stichotrichia	Urostylida	Urostylidae	***Neourostylopsis flavicana***	Lab of Protozoology	FJ775718	JQ424869	JQ424857	KM222028
152	SPIROTRICHEA	Stichotrichia	Urostylida	Urostylidae	***Parabirojimia multinucleata***	Lab of Protozoology	FJ156104	GQ246482	KM222129	GQ258108
153	APICOMPLEXA				***Theileria parva***	GenBank	L02366	U03602	AF013419	XM_758635
154	APICOMPLEXA				***Eimeria tenella***	GenBank	AF026388	AF026388	AF026388	HG675723
155	APICOMPLEXA				***Toxoplasma gondii***	GenBank	X75429	X75429	X75429	XM_002364766
156	DINOPHYCEAE				***Perkinsus marinus***	GenBank	AF497479	AF497479	AF497479	AF482399
157	DINOPHYCEAE				***Karenia brevis***	GenBank	EF492502	FJ823562	AY355459	AF482419
158	DINOPHYCEAE				***Alexandrium tamarense***	GenBank	JF521639	JF521639	JF521639	NA

Supporting information

Figure S1. Maximum likelihood (ML) tree focusing on 113 ciliate representatives based on 5.8S rDNA sequences.

Figure S2. Bayesian inference (BI) tree focusing on 113 ciliate representatives based on 5.8S rDNA sequences.

Figure S3. Maximum likelihood (ML) tree focusing on 116 ciliate representatives based on alpha-tubulin amino acid sequences.

Figure S4. Bayesian inference (BI) tree focusing on 116 ciliate representatives based on alpha-tubulin amino acid sequences.

Figure S5. Maximum likelihood (ML) tree focusing on 116 ciliate representatives based on alpha-tubulin first two codon positions.

Figure S6. Bayesian inference (BI) tree focusing on 116 ciliate representatives based on alpha-tubulin first two codon positions.

Figure S1. Maximum likelihood (ML) tree focusing on 113 ciliate representatives based on 5.8S rDNA sequences.

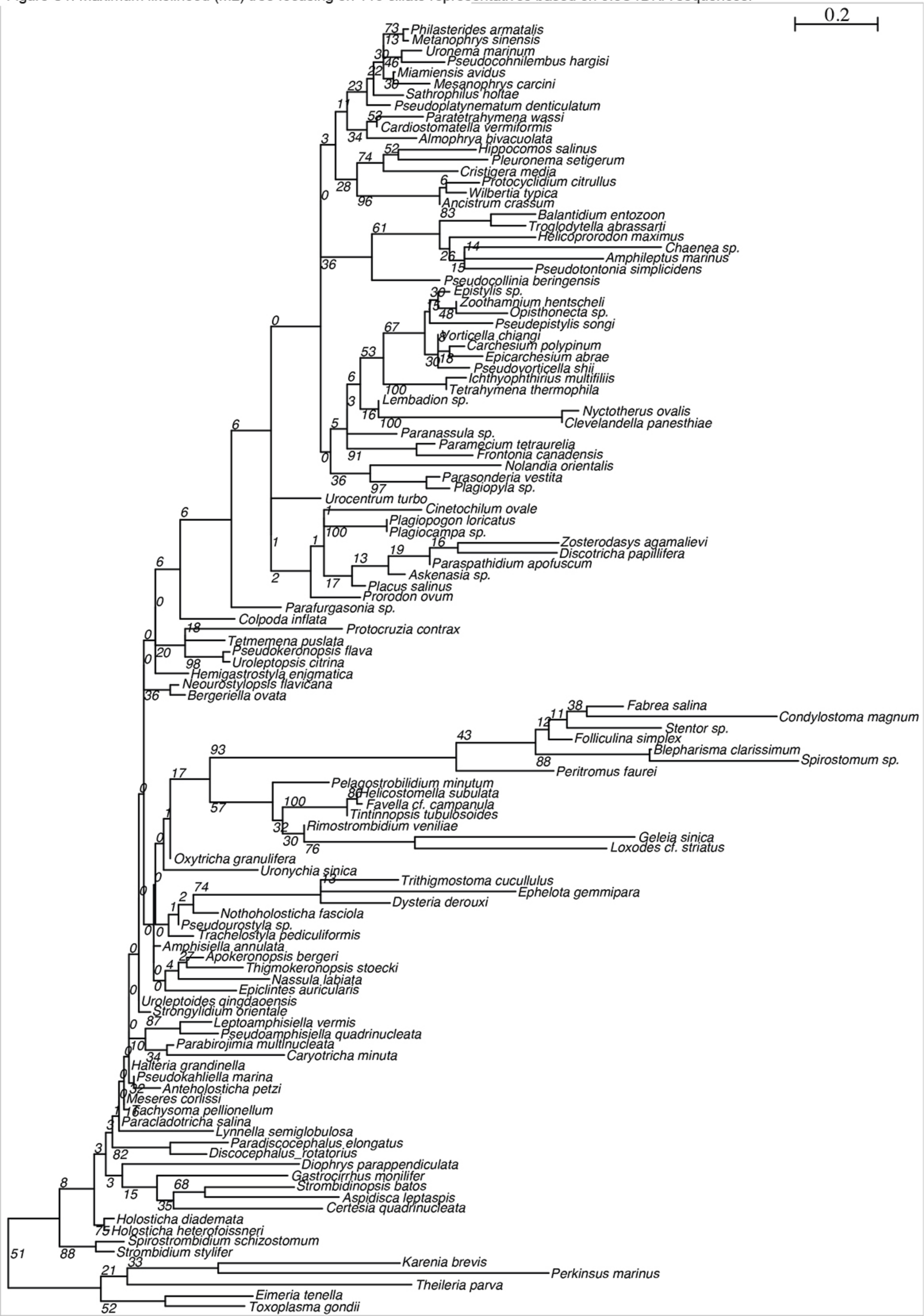

Figure S2. Bayesian inference (BI) tree focusing on 113 ciliate representatives based on 5.8S rDNA sequences.

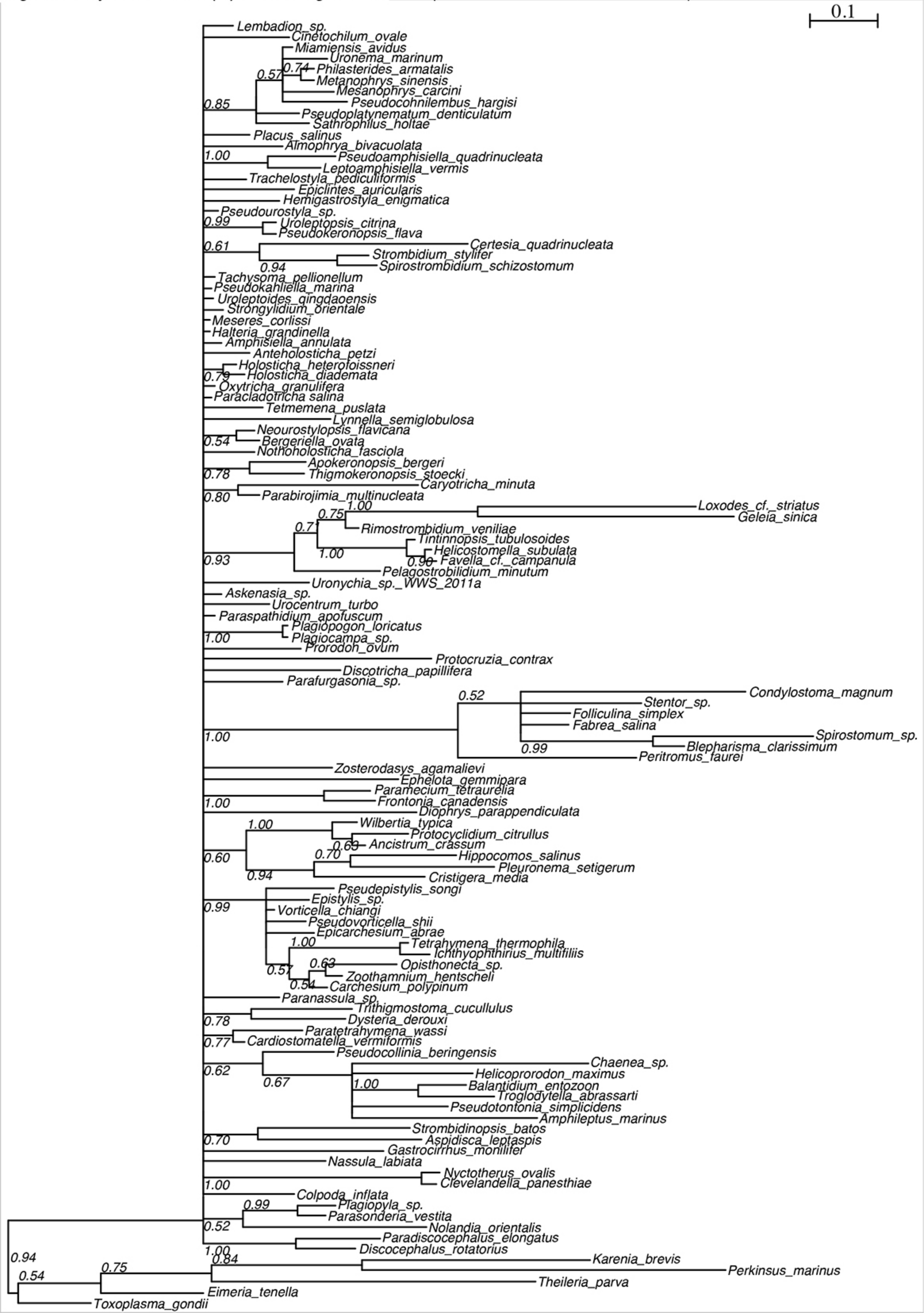

Figure S3. Maximum likelihood (ML) tree focusing on 116 ciliate representatives based on alpha-tubulin amino acid sequences.

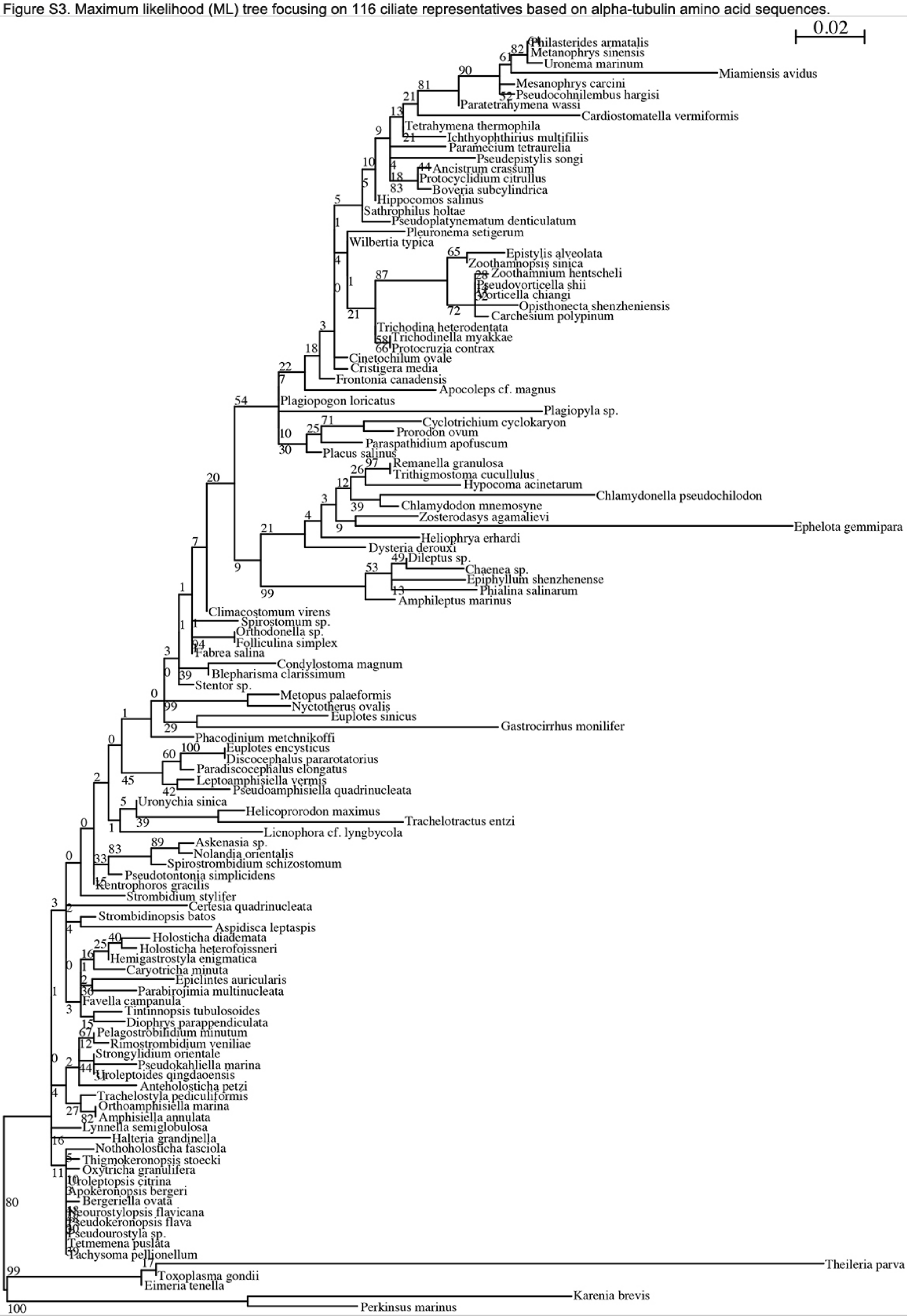

Figure S4. Bayesian inference (BI) tree focusing on 116 ciliate representatives based on alpha-tubulin amino acid sequences.

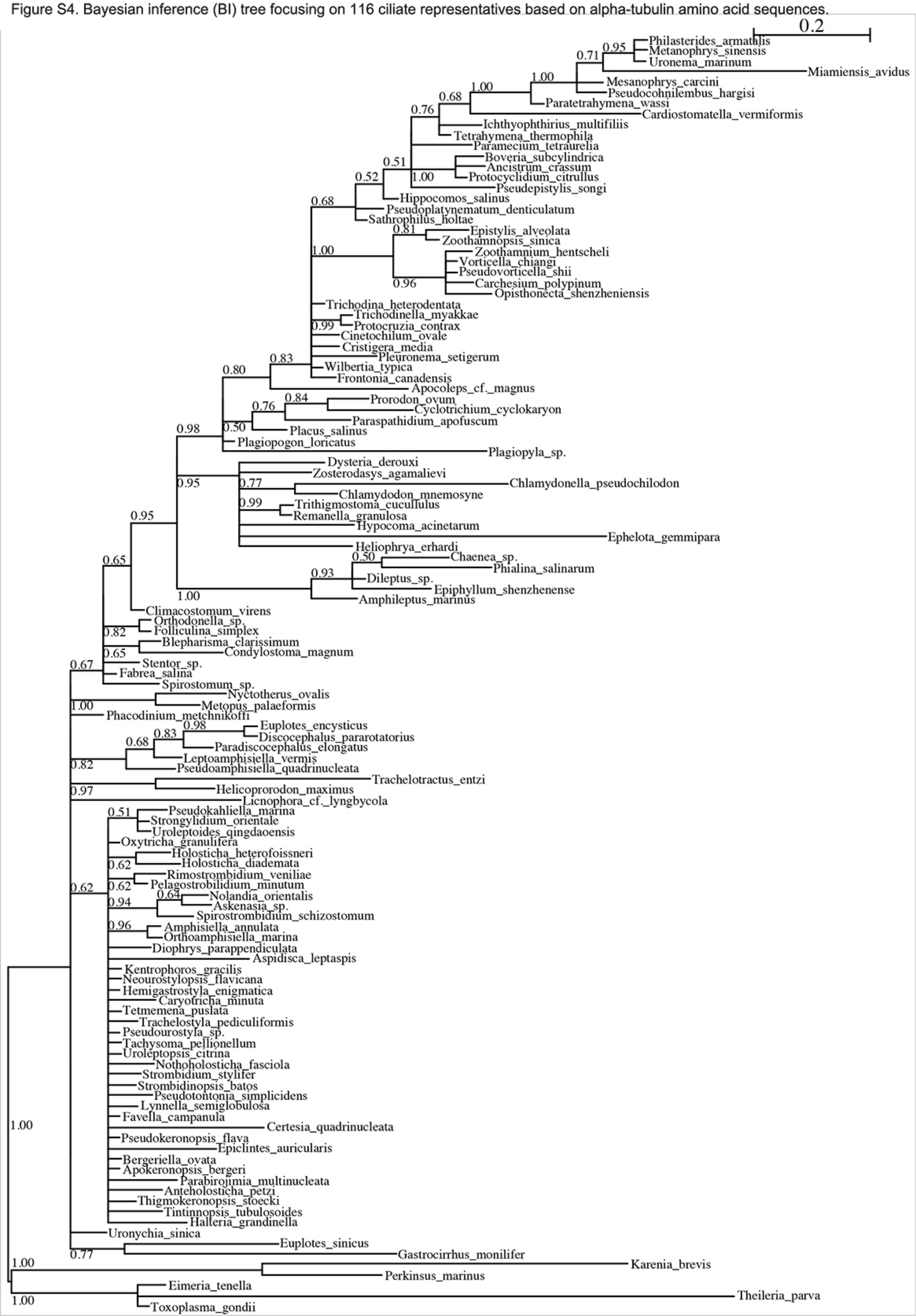

Figure S5. Maximum likelihood (ML) tree focusing on 116 ciliate representatives based on alpha-tubulin first two codon positions.

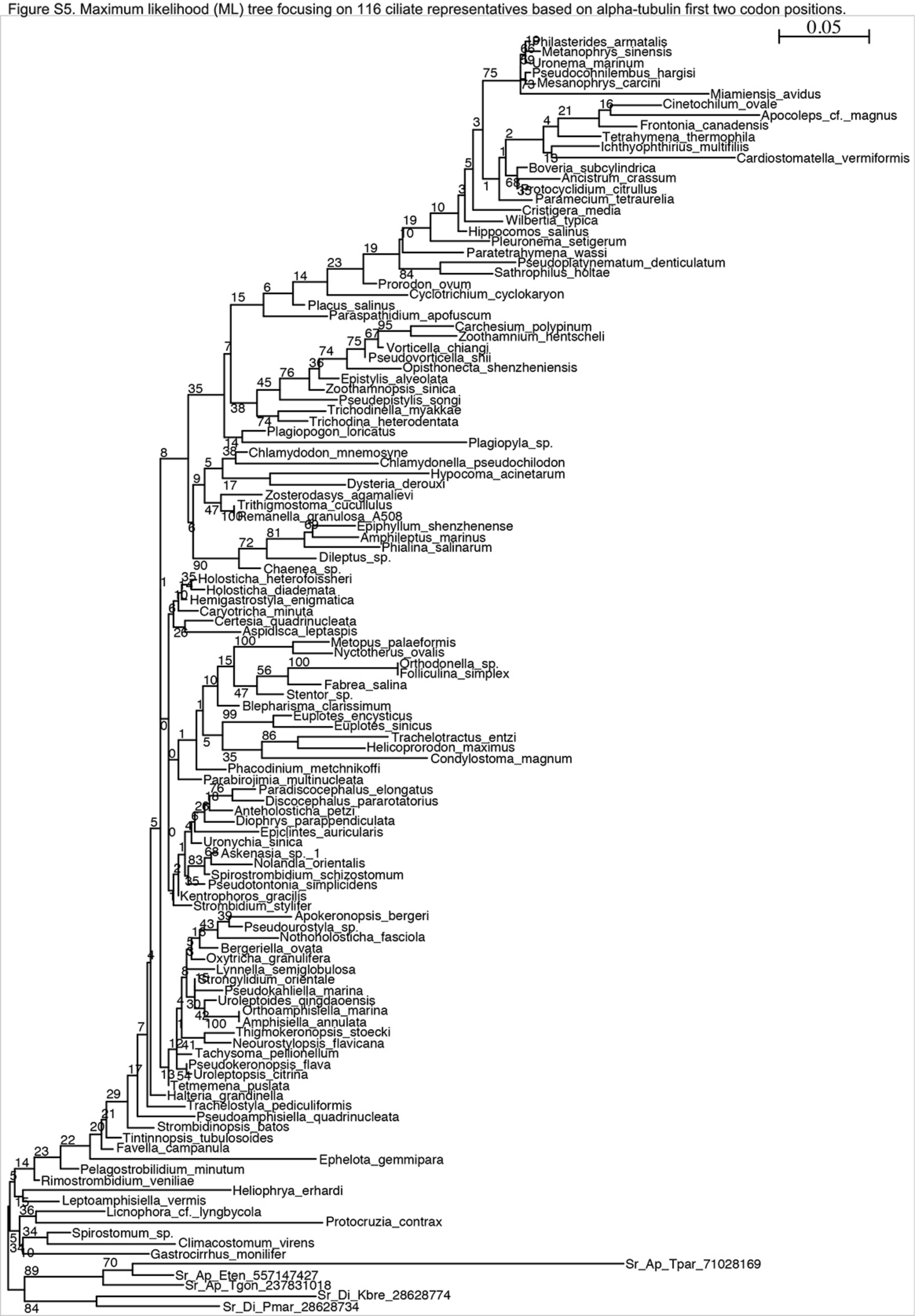

Figure S6. Bayesian inference (BI) tree focusing on 116 ciliate representatives based on alpha-tubulin first two codon positions

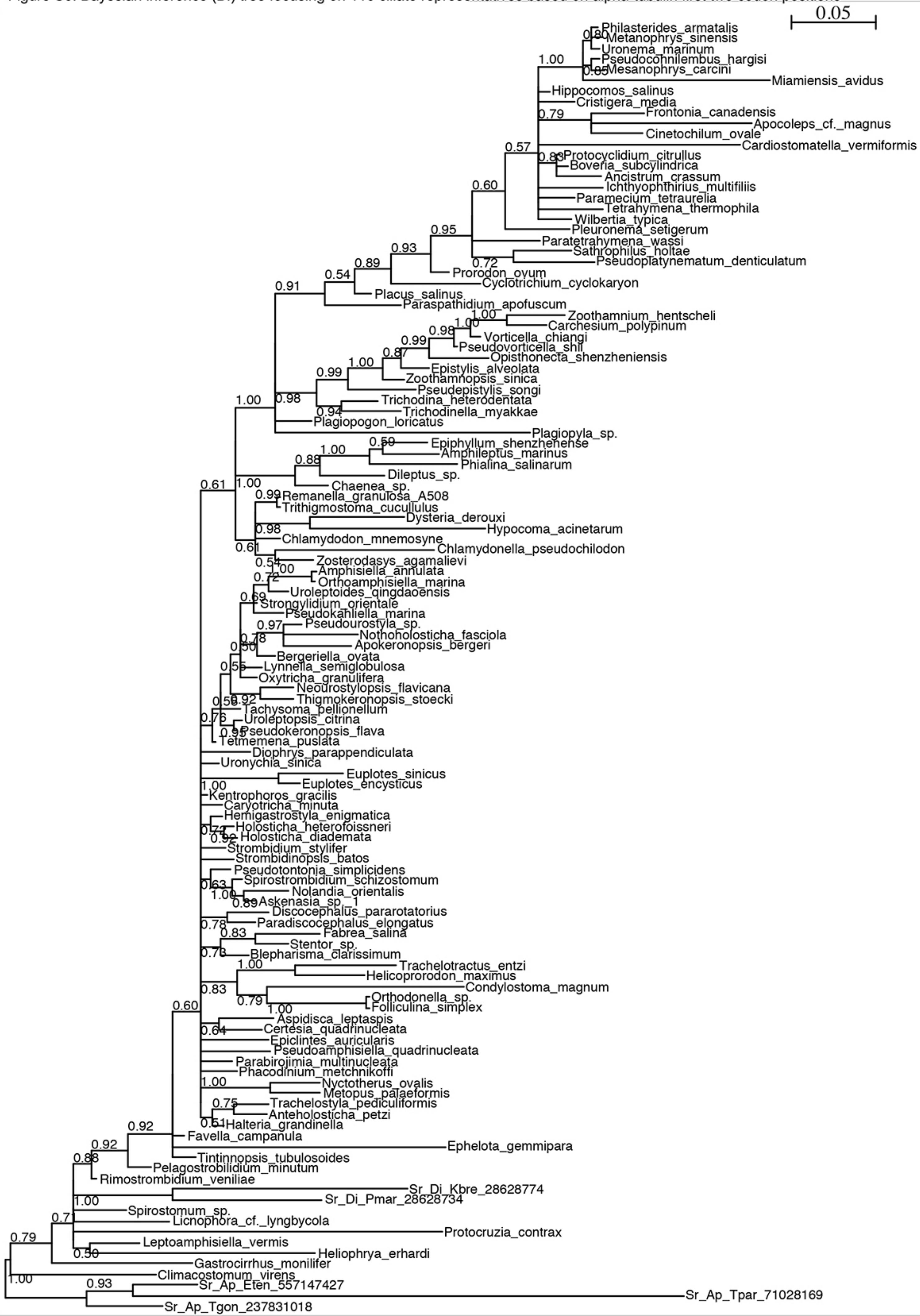

Supplement: Supplementary Information [file srep24874-s1.pdf]
